# First complete mitochondrial genome of the South American annual fish *Austrolebias charrua* (Cyprinodontiformes: Rivulidae): peculiar features among cyprinodontiforms mitogenomes

**DOI:** 10.1186/s12864-015-2090-3

**Published:** 2015-10-28

**Authors:** Verónica Gutiérrez, Natalia Rego, Hugo Naya, Graciela García

**Affiliations:** Sección Genética Evolutiva, Facultad de Ciencias, Universidad de la República, Iguá 4225 (CP.11400), Montevideo, Uruguay; Institut Pasteur de Montevideo, Unidad de Bioinformática, Montevideo, Uruguay; Departamento de Producción Animal y Pasturas, Facultad de Agronomía, Universidad de la República, Paysandú, Uruguay

**Keywords:** High-throughput sequencing, *Austrolebias charrua*, Cyprinodontiformes, Comparative mitogenomics, Molecular markers

## Abstract

**Background:**

Among teleosts, the South American genus *Austrolebias* (Cyprinodontiformes: Rivulidae) includes 42 taxa of annual fishes divided into five different species groups. It is a monophyletic genus, but morphological and molecular data do not resolve the relationship among intrageneric clades and high rates of substitution have been previously described in some mitochondrial genes. In this work, the complete mitogenome of a species of the genus was determined for the first time. We determined its structure, gene order and evolutionary peculiar features, which will allow us to evaluate the performance of mitochondrial genes in the phylogenetic resolution at different taxonomic levels.

**Results:**

Regarding gene content and order, the circular mitogenome of *A. charrua* (17,271 pb) presents the typical pattern of vertebrate mitogenomes. It contains the full complement of 13 proteins-coding genes, 22 tRNA, 2 rRNA and one non-coding control region. Notably, the tRNA-Cys was only 57 bp in length and lacks the D-loop arm. In three full sibling individuals, heteroplasmatic condition was detected due to a total of 12 variable sites in seven protein-coding genes. Among cyprinodontiforms, the mitogenome of *A. charrua* exhibits the lowest G+C content (37 %) and GCskew, as well as the highest strand asymmetry with a net difference of T over A at 1st and 3rd codon positions. Considering the 12 coding-genes of the H strand, correspondence analyses of nucleotide composition and codon usage show that A and T at 1st and 3rd codon positions have the highest weight in the first axis, and segregate annual species from the other cyprinodontiforms analyzed. Given the annual life-style, their mitogenomes could be under different selective pressures. All 13 protein-coding genes are under strong purifying selection and we did not find any significant evidence of nucleotide sites showing episodic selection (dN >dS) at annual lineages. When fast evolving third codon positions were removed from alignments, the “supergene” tree recovers our reference species phylogeny as well as the Cytb, ND4L and ND6 genes. Therefore, third codon positions seem to be saturated in the aforementioned coding regions at intergeneric Cyprinodontiformes comparisons.

**Conclusions:**

The complete mitogenome obtained in present work, offers relevant data for further comparative studies on molecular phylogeny and systematics of this taxonomic controversial endemic genus of annual fishes.

**Electronic supplementary material:**

The online version of this article (doi:10.1186/s12864-015-2090-3) contains supplementary material, which is available to authorized users.

## Background

The Neotropical genus *Austrolebias* (Cyprinodontiformes: Rivulidae) is a locally endemic group of annual fishes. It is distributed from northern and northeastern Argentina, Paraguay, Uruguay and southern Brazil throughout the Paraná-Plata basin and Patos-Merín system [1]. It includes 42 known species (http://www.fishbase.org/search.php) highly variable in morphology and behavior [2, 3]. They live in temporary ponds formed during rainy seasons where each generation completes full life cycle within 1 year. The population survives dry season in the form of eggs buried in the mud. During the subsequent rainy season, the ponds refill, the eggs hatch and the larvae rapidly grow to sexual maturity and reproduce [4, 5]. According to morphological and molecular characters these taxa are divided into five species groups: *A. adloffi*, *A. alexandri*, *A. bellottii*, *A. elongatus* and *A. robustus* [1, 6, 7]. Molecular phylogenetic analyses based on some mitochondrial genes, supported the monophyly of the genus, but exhibited limited resolution at deeper nodes of the trees. This lack of information could be due to a saturation of nucleotide changes in the genes analyzed and/or to the mode of evolutionary diversification of the *Austrolebias* taxa involved [6, 8]. High levels of intrageneric sequence divergence and saturation have been detected in the cytochrome b gene, and a bias in favor of AT changes has been detected at silent sites in this gene [6]. As proposed by Martin and Palumbi [9], high rates of nucleotide substitutions observed in mitochondrial genes could be related to some physiological variables such as generation time, life span, age at first reproduction, rate of population increase, and metabolic rate. In fact, high rates of growth were found related to an increase of temperature up to 25 °C in natural and laboratory populations of *A. viarius* [10].

In the present work we describe for the first time the complete mitochondrial genome of *A. charrua* (Costa and Cheffe 2001), a member of the *A. adloffi* species group, highlighting its structural, compositional and evolutionary features. While a wealth of data on mtDNA sequence and gene organization of fish mitogenomes have been collected in the last years, this is the first reference mitogenome for the genus, and just the second within the Rivulidae family [11]. To date, 1750 mtDNA sequences (update June 2, 2015) are deposited in the Mitochondrial Genome Database of Fish (MitoFish: http://mitofish.aori.u-tokyo.ac.jp/), of which 15 correspond to Cyprinodontiformes. We took advantage of some of these available mitogenomes and compared them in an evolutionary framework, searching for putative molecular patterns associated to the annual life style. For the different mitochondrial coding regions, we also evaluated their performance as molecular markers for phylogenetic reconstruction at different taxonomic levels.

## Methods

### Sample collection and mtDNA extraction

A total of three individuals of *A. charrua* (AUS4, AUS5 and AUS7) were analyzed in the present work. They belonged to the same F1 generation obtained at the lab while the parents were collected in a temporal pond located in “La Coronilla” (Rocha Department, Uruguay). AUS4 and AUS5 were young individuals of 3 months old, while AUS7 was an adult of 5 months old. All tissues and voucher specimens were deposited in the Sección Genética Evolutiva, Facultad de Ciencias, Universidad de la República, Montevideo, Uruguay.

The mtDNA was isolated from muscle tissue (fixed in 95 % ethanol) of freshly sacrificed animals by an over-exposure to a solution of 1‰ 2-phenoxyethanol (Sigma). The sampling protocol was approved by the CNEA (Comisión Nacional de Experimentación Animal) from Uruguay.

### Sequencing

Sequencing libraries were generated using Nextera XT kit (Illumina, USA) from 1 ng of input DNA. Tagmentation of genomic DNA, PCR amplification (12 cycles) with addition of index primers and library normalization, were performed according to manufacturer’s protocol. Quality and length of libraries were assessed with Agilent High Sensitivity DNA Kit (Agilent, USA) using the 2100 Bioanalyzer (Agilent, USA) before the final normalization step. Multiplexed libraries were sequenced on an Illumina Genome Analyzer IIx platform run for 66 cycles in single-end mode, at the Institut Pasteur of Montevideo.

### Amplification and sequencing of the control region

Amplification of the control region was performed by PCR. Primers were designed at both ends of the automatically assembled CR (tRNA-Pro and tRNA-Phe). Two sets of specific primers: ACR-1F (5′- ACC TGT TCC TCT AGC ACC CA -3′), ACR-1R (5′- TGT AGG AGG CAT TTA AGG TGC A -3′) and ACR-2F (5′- TTT CTG GCC CAC AAG AGA CC -3′), ACR-2R (5′- TGC TCA TGA AAC TTT TTA GGG TTT -3′) were created using Primer3 v. 0.4.0 software. The PCR was carried out in a 10 μL total volume of 10× buffer, MgCl_2_ 2.5 mM, dNTPs 0.2 mM, 0.4 mM of each primer, 0.5 U of *Taq* DNA polymerase (Invitrogen) and 10 ng of DNA, under the following conditions: one denaturation step at 94 °C for 5 min, followed by 35 cycles of 94 °C for 45 s, 62 °C for 45 s and 72 °C for 1 min, and a final elongation step at 72 °C for 7 min.

Sequencing reactions were performed on each template on the automated ABI PRISM 377 DNA Sequencer (MACROGEN Inc., Korea).

### Mitogenome assembly and annotation

Raw reads were quality filtered and trimmed to a final length of at least 50 bp. As indicated previously [12], small mitochondrial genomes are present in variable copy numbers in total DNA mixed samples, therefore transcriptome-designed tools are better suited for mitogenome assembly. Following [12], contigs were assembled using SOAPdenovo-Trans [13] with k-mer length of 31 and remaining default options. As both nuclear and mitochondrial contigs were assembled with this strategy, a local blastn [14] was ran against a database of cyprinodontiforms mitogenomes downloaded from MitoFish [15] (MitoFish version 2.89, update March 2014, 1361 complete mitochondrial sequences), to recover those contigs of mitochondrial origin.

Selected contigs were annotated with MitoAnnotator [15], a highly accurate and automated pipeline specific for fish mitogenomes, followed by manual curation in Artemis [16]. Transfer RNA genes were annotated based on their potential cloverleaf secondary structure using MiTFi [17], a tool for accurately locating tRNA genes within mitogenomic sequences (MiTFi was run through MitoAnnotator [15]).

### Variant analysis

Reads from each sample were mapped to the chosen reference mitogenome in order to search for the presence of nucleotide substitutions and indels within mitogenomes (i.e. to search for heteroplasmy, defined as the presence of more than one type of mitogenome within one individual) and among siblings. This was performed with a combination of Burrows-Wheeler Aligner (bwa; default options) [18] and SAMtools (samtools view with -q 30; samtools mpileup with options -B -C 0 -Q 30) [19]. Of note, both the read mapping quality (−q 30) and base quality (−Q 30) were required to have a minimum value of 30 in a Phred scoring scheme to be considered in the variant analysis.

### Compositional analyses

Nucleotide composition, as well as codon and amino acid usage were calculated in the R environment [20] using seqinR [21] and ca packages [22]. Besides whole mitogenome analyses, calculations were performed on multiple alignments of the 12 protein-coding genes present in the H strand (ND6 was excluded due to its location in the L strand where there are different compositional biases). In these comparative analyses, the following Cyprinodontiformes were included: *Kryptolebias marmoratus* (Aplocheiloidei: Rivulidae), *Nothobranchius furzeri* (Aplocheiloidei: Nothobranchiidae), *Aplocheilus panchax* (Aplocheiloidei: Aplocheilidae), *Cyprinodon rubrofluviatilis* (Cyprinodontoidei: Cyprinodontidae) and *Fundulus olivaceus* (Cyprinodontoidei: Fundulidae). To note, while *K. marmoratus* is the closest relative to *A. charrua*, *N. furzeri* is the only annual killifish other than *A. charrua* in this species sample. *Cyprinodon rubrofluviatilis* and *F. olivaceus* were considered outgroup lineages. The phylogenetic tree for these taxa according to Pohl et al. [23] topology is shown in Additional file 1. Multiple nucleotide alignments were built guided by previous alignments of amino acid sequences using Muscle [24] and Pal2Nal [25]. For each alignment, ancestral sequence reconstruction was performed with a joint-likelihood reconstruction method in the codon-state space [26, 27].

### Protein-coding evolutionary analyses

Analyses of signatures of positive selection on codon sequences were carried out using the suite of routines implemented in http://www.datamonkey.org [28]. Uploaded alignments were matched with the reference species tree (Additional file 1) and *F. olivaceus* fixed as outgroup lineage if required. Global dN/dS values were estimated with SLAC [27]. MEME [29] and BS-REL [30] models were used to search sites and branches under episodic and pervasive selection. For MEME, sites were posited to be under directional selection at selected branches if they: i) showed non-synonymous to sysnonymous substitution rate β+ >1; ii) were significant at α = 0.05 level; and iii) also showed an Empirical Bayes Factor >20. Both MEME and BS-REL avoid the *a priori* and rigid partitioning of branches into “foreground” and “background” classes. BS-REL uses a random effects likelihood framework, in which ω can take one of three values along branches (ωb- ≤ωbN ≤1 ≤ωb+) to explore every branch-site combination. Sequential likelihood ratio testing is used to identify branches with significant amounts of diversifying selection.

### Phylogenetic reconstruction

Nucleotide alignments for individual protein-coding genes were used to infer maximum likelihood phylogenies with PhyML v3.1 [31]. Also, a “supergene” phylogeny was based on a concatenated alignment of all 13 protein-coding genes. In each case, five random starting trees were used to search the tree space by NNI [32] and SPR [33] methods. Survey of branch support was conducted with a Bayesian-like transformation of approximate likelihood ratio test (aBayes; [34]). Nucleotide substitution model parameters were determined according to the best suited sequence substitution model selected by ModelGenerator v0.85 [35], following Bayesian information criterion. For most genes, the HKY+I+G model [36] was the chosen one, while the GTR+I+G [37] suited best for COI, COIII and the concatenated alignment. Default values were kept for remaining PhyML options. Inferred gene trees were visualized to study their concordance to the working species tree hypothesis (Additional file 1). All inferences were recalculated after third codon positions were removed from the alignments (Additional file 2).

## Results and discussion

### *Austrolebias charrua* mitogenome

The mtDNA sequence of the annual fish *A. charrua* was determined by massively parallel sequencing methods. After the quality filtered and trimmed reads were assembled with SOAPdenovo-trans [13], almost the whole mitochondrial genome was reconstructed in only one contig in the samples AUS4 and AUS7, while two contigs were assembled in case of AUS5 (all contigs reached mean coverage above 30×; Table 1). The mitochondrial control region is a non-coding element vital for the initiation of both mtDNA replication and transcription. Often, it contains repetitive elements and could appear as a duplicated region as shown in the mitogenome of the closely related non-annual rivulid species *K. marmoratus* [11]. These features pose some difficulties during the automatic assembly of mitogenomes where duplicated elements are prone to be collapsed in the final assembly. Therefore, Sanger sequencing was used to confirm the size and sequence of the control region. Eight CR sequences obtained from the samples AUS1 and AUS7, revealed that it spans from the tRNA-Pro to the tRNA-Phe and is 1349 bp long, showing an internal duplication of 180 bp which had not been resolved during the automatic assembly (Additional file 3).

**Table 1:** Illumina sequencing and SOAPdenovo-trans assembly

	AUS4	AUS5	AUS7
#raw reads	9490242	6917778	5580271
#final reads	8941178	5015221	3659292
#contigs with mitochondrial sequences^a^	1	2	1
Contig length	17034	5341;11724	17103
Contig mean coverage	42	46;51	31

^a^Similarity was determined by blastn search against a database of mitogenomes of Cyprinodontiformes; the four contigs showed above 73 % similarity and E-value close to zero

**Fig. 1:**
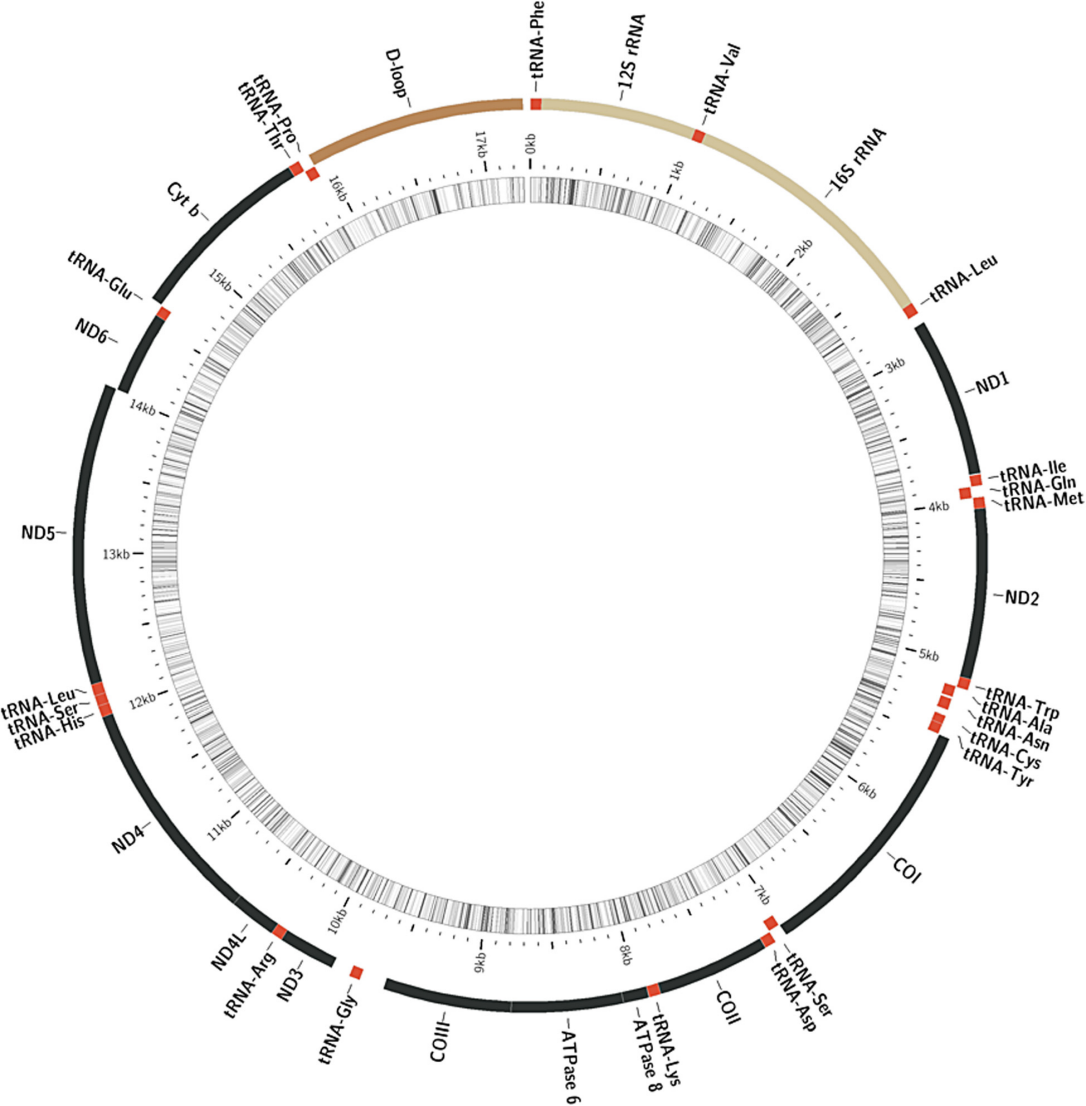
Map of the mitochondrial genome of *Austrolebias charrua* (GenBank accession number KP718940). Visual representation created by Circos [51] where the innermost circle represents G+C content per every 5 bp (darker lines are the higher G+C content) and the external one represents the two mtDNA strands. Protein-coding genes are in black; tRNAs in red and are designated by their three letter abbreviations; light brown are the rRNAs and in dark brown is the CR (D-loop)

**Fig. 2:**
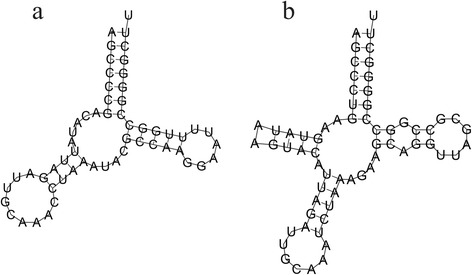
Putative secondary structure of the tRNA-Cys encoded in the mtDNA of (**a**) *A. charrua* and (**b**) *K. marmoratus*. The tRNAs were annotated using MiTFi tool run through MitoAnnotator [15]

The *de novo* assembled mitochondrial contig of AUS7 was manually curated and edited using this additional sequence information. The resulting complete mitochondrial sequence of 17,271 bp (the initial contig was 17,103 bp) was deposited in the GeneBank (accession number KP718940) and considered our reference mitogenome for *A. charrua* (Fig. 1). Regarding gene content and order, it exhibits the typical pattern of vertebrate mitogenomes [38]. It contains the full complement of 13 protein-coding genes, 22 tRNA genes, two rRNA genes (12S and 16S) and one non-coding control region. Eight tRNAs (tRNA-Gln, tRNA-Ala, tRNA-Asn, tRNA-Cys, tRNA-Tyr, tRNA-Ser(1), tRNA-Glu and tRNA-Pro) and the protein-coding gene ND6 were encoded by the light (L) strand, whilst the remaining genes were encoded by the heavy (H) strand (Table 2). ATG codons initiate 12 of the protein-coding genes, COI being the exception (GTG start codon). Complete TAA stop codons are present in COI, COIII, ATPase8, ND1, ND4L, ND5 and Cytb, while a complete TAG codon is present in ND6. Remaining protein genes end at incomplete codons (i.e. T or TA), which are converted by polyadenylation into TAA after transcription and processing [39]. Of 22 transfer RNA genes, 21 were within the size range of 66–74 bp (Table 2), and each of them folded into a typical cloverleaf secondary structure. On the other hand, tRNA-Cys was only 57 bp in length, shorter than any known annotated tRNA in Cyprinodontiformes (z-score = −5.74) and in the predicted secondary structure lacks the D-loop arm (Fig. 2a). While D-armless tRNA-Cys reports do not exist for fishes [17], several independent occurrences do exist in metazoans, including mammals. Atypical tRNAs (D or T-armless) would be functional [17, 40, 41]. To note, in *K. marmoratus*, tRNA-Cys is 65 bp in length and has the typical secondary structure (Fig. 2b). Further analysis of new *Austrolebias* taxa mitogenomes could clarify whether this D-armless tRNA-Cys is a genus or species-level feature.

**Table 2:** Organization of the mitochondrial genome of *Austrolebias charrua*

	Location		Size		Codon			
Gene	Start	End	Nucleotide (bp)	Amino acid	Start	Stop	Intergenic nucleotide (bp)	Strand
tRNA-Phe	1	69	69					H
12S rRNA	70	1016	947				0	H
tRNA-Val	1017	1084	68				0	H
16S rRNA	1085	2715	1631				0	H
tRNA-Leu(1)	2716	2789	74				0	H
*ND1*	2854	3828	975	324	ATG	TAA	64	H
tRNA-Ile	3835	3902	68				6	H
tRNA-Gln	3902	3972	71				−1	L
tRNA-Met	3972	4040	69				−1	H
*ND2*	4041	5085	1045	348	ATG	T--	0	H
tRNA-Trp	5086	5153	68				0	H
tRNA-Ala	5152	5221	70				−2	L
tRNA-Asn	5234	5306	73				12	L
tRNA-Cys	5343	5399	57				36	L
tRNA-Tyr	5399	5464	66				−1	L
*COI*	5466	7022	1557	518	GTG	TAA	1	H
tRNA-Ser(1)	7026	7096	71				3	L
tRNA-Asp	7100	7168	69				3	H
*COII*	7170	7860	691	230	ATG	T--	1	H
tRNA-Lys	7861	7932	72				0	H
ATPase 8	7934	8101	168	55	ATG	TAA	1	H
ATPase 6	8092	8774	683	227	ATG	TA-	−10	H
*COIII*	8775	9560	786	261	ATG	TAA	0	H
tRNA-Gly	9711	9778	68				150	H
*ND3*	9894	10242	349	116	ATG	T--	115	H
tRNA-Arg	10243	10311	69				0	H
*ND4L*	10312	10608	297	98	ATG	TAA	0	H
*ND4*	10602	11991	1390	463	ATG	T--	−7	H
tRNA-His	11992	12060	69				0	H
tRNA-Ser(2)	12061	12127	67				0	H
tRNA-Leu(2)	12127	12198	72				−1	H
*ND5*	12199	14043	1845	614	ATG	TAA	0	H
*ND6*	14040	14561	522	173	ATG	TAG	−4	H
tRNA-Glu	14562	14629	68				0	L
*Cytb*	14634	15782	1149	382	ATG	TAA	4	H
tRNA-Thr	15784	15853	70				1	H
tRNA-Pro	15853	15922	70				−1	L
Control region	15923	17271	1349				0	H

Standard abbreviations are used for protein-coding genes and three letter abbreviations are given for tRNAs and rRNAs. Intergenic nucleotide refers to the nucleotide distance between pairs of adjacent genes

### Mitogenome differences among full siblings

The availability of deep sequencing data allows the discovery of hidden variation and heteroplasmy. Reads were mapped against the assigned *A. charrua* reference genome (AUS7) and mpileup files generated as mentioned in methods. R scripts [20] and careful inspection with IGV [42] were used to determine the presence of nucleotide variants. For those mitogenome positions where the mapping coverage was at least 20×, a variant was defined if: i) there was only one alternative nucleotide; ii) the alternative base showed up in at least two reads, with at least one read mapping to each DNA strand (this requirement is aimed to discard sequencing errors linked to specific biases in DNA composition as well as spurious support due to existence of duplicated reads); and iii) alternative nucleotides were far away from the read ends and the neighborhood positions were error-free. These requirements were defined after joint consideration of reviewed references [43–47] and the features (e.g. mean sequencing coverage) of our data. A total of 12 variants were detected in protein-coding genes of the mitogenome of the full siblings analyzed, five in the mtDNA of AUS4 and seven in AUS5. The diversity of changes and their putative effects on the coding sequence are shown in Table 3. The transversion C to A identified in the COI gene (position 6245) was present in both samples, while the transition T to C detected in the Cytb gene (position 15,735) was present in all full siblings, including the reference genome (Table 3). The coverage depth of this experiment would not allow the detection of *de novo* mutations, so the variants observed would be all putative changes already carried in the maternal mitogenomes which had changed their frequency in the different siblings as consequence of sampling effects [44, 46]. To note, above 28 % of the reads mapping to position 15,735 carried the alternative nucleotide cytosine. This heteroplasmic position corresponds to the codon 368 of the Cytb locus, and despite of being a transition it produces a non-synonymous change from Phe to Leu in the translated sequence (TTT to CTT codon change). Phe and Leu are both non-polar amino acids but the change is from an aromatic residue to a non-aromatic one. However, hydrophobic aromatic amino acids can be sometimes substituted by aliphatic residues of a similar size, as in the present case. In addition, two related findings preclude considering this variant as having a notorious phenotype in the organism. First, given a multiple alignment of metazoan Cytb sequences, the position is highly variable, being Leu the consensus amino acid (Additional file 4). Second, the involved residue is part of the H helix in the intermembrane domain, close to the C-terminus of the protein. This is one of the least conserved eight helices in the protein, lacks heme binding-sites and does not interact with other components of the cytochrome bc1 complex [48, 49].

**Table 3:** Nucleotide variants in the mitogenomes of three full siblings of *A. charrua*

#position	Gene	Variant and total reads per individual			Nucleotide change	Ts or Tv	#codon	Codon change	Aminoacid change	Effect
		AUS4	AUS5	AUS7						
4105	ND2		2/167		C to A	Tv	22	TCG to TAG	Ser to STOP	Nonsense
5690	COI	2/74			T to A	Tv	75	ATT to AAA	Ile to Met	Non-synonymous
6245	COI	2/153	3/171		C to A	Tv	260	TAC to TAA	Tyr to STOP	Nonsense
7561	COII		2/109		G to T	Tv	131	GGC to GTC	Gly to Val	Non-synonymous
10220	ND3	2/151			A to G	Ts	109	CAA to CAG	Gln to Gln	Synonymous
11804	ND4	2/151			G to T	Tv	401	GGG to GGT	Gly to Gly	Synonymous
12505	ND5		2/191		T to C	Ts	103	TCT to CCT	Ser to Pro	Non-synonymous
12764	ND5		2/108		C to T	Ts	189	GCC to GCT	Ala to Ala	Synonymous
12962	ND5		2/85		G to T	Tv	255	AGC to ATC	Ser to Ile	Non-synonymous
15735	Cytb	44/93	35/90	15/53	T to C	Ts	368	TTT to CTT	Phe to Leu	Non-synonymous

Reads were mapped against the reference genome AUS7. The number of reads carrying the variant is shown together with total number the reads mapping to each position (variant reads/total reads)

### Comparative mitogenomics of Cyprinodontiformes: structural and evolutionary features

**Fig. 3:**
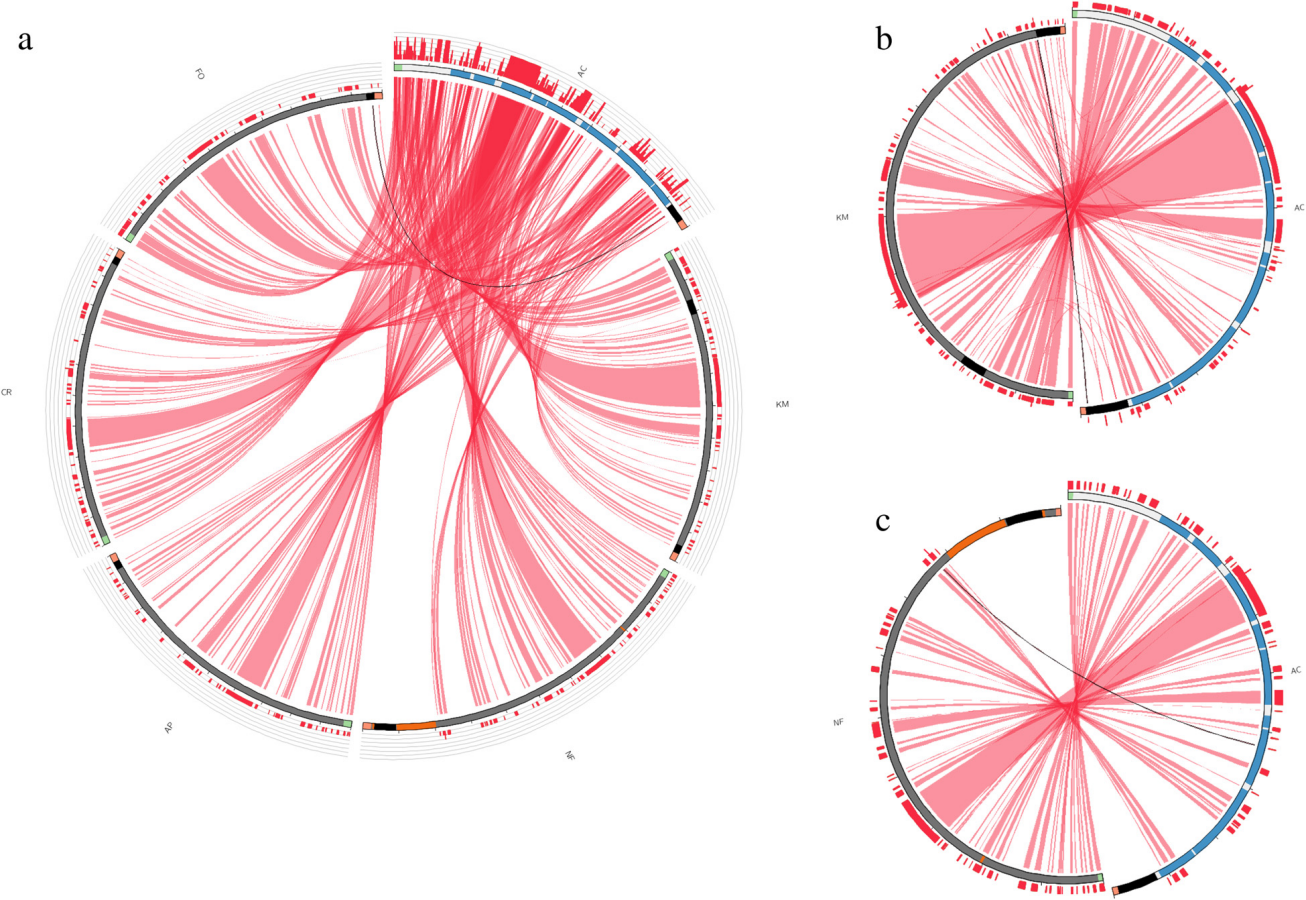
Circos plot mapping the synteny and identity level of *A. charrua* mitogenome against each of the other five mitogenomes studied (**a**), *K. marmoratus* (**b**) and *N. furzeri* (**c**). Idiograms and *red ribbons* represent the similarity after pairwise blastn searches. In *A. charrua* idiogram, the 13 coding genes are coloured in *blue*. In all idiograms, control region are coloured in *black*. In (**c**), *N. furzeri* unique features (tRNA-Gln, tRNA-Glu and the interpersed region between tRNA-Thr and tRNA-Pro) are shown in *orange*. The figure was produced using Circos software [51], a tool for graphical representation of genome data, through the Circoletto implementation [88]. AC (*A. charrua*), KM (*K. marmoratus*), NF (*N. furzeri*), AP (*A. panchax*), CR (*C. rubrofluviatilis*) and FO (*F. olivaceus*)

The whole mitogenomes compared in present work exhibit a similar total length, but few differences have been detected regarding the CR (Table 4). *Kryptolebias marmoratus* displays an additional control region located between tRNA-Leu and ND1 [11] while the annual species *N. furzeri* shows a more complex rearrangement where a duplication of tRNA-Gln sites in place of tRNA-Glu, which in turn is located in the L strand between the control region and the tRNA-Phe. Besides, a long intergenic region is interspersed between tRNA-Thr and tRNA-Pro [50]. A Circos plot [51] highlighting synteny and identity level between *A. charrua* and each of the five mitogenomes used for comparison is shown in Fig. 3. The most notorious feature in the plot is the lack of similarity between *A. charrua* and the last region of *N. furzeri* mitochondrial chromosome (about 3500 pb), despite homologous regions can be identified in more distant species as *C. rubrofluviatilis* and *F. olivaceous* (Figs. 3a, c). Considering that the mitogenome of *N. furzeri* was built as part of a whole genome shotgun approach resulting in a narrow 5.4 Mb draft assembly (out of an estimated 1.59 – 1.92 Gb total genome size) [52] a putative miss-assembly should be discarded before further comparative analyses.

**Table 4:** Nucleotide composition of all Cyprinodontiformes compared in present work

Species	GenBank accession number	Total length (bp)	CR length (bp)^a^	A	C	G	T	GCskew	ATskew	GC1	GC2	GC3	Coding GC
*Austrolebias charrua* ^b^	KP718940	17121	1349	0.284	0.222	0.146	0.348	−0.207	−0.102	44.980	38.670	28.770	0.375
*Nothobranchius furzeri* ^b^	NC011814	19527	2091	0.312	0.236	0.149	0.302	−0.225	0.016	44.840	39.360	31.290	0.385
*Kryptolebias marmoratus*	NC003290	17329	887	0.275	0.275	0.158	0.292	−0.270	−0.029	49.510	40.200	41.620	0.438
*Aplocheilus panchax*	NC011176	16519	860	0.292	0.269	0.158	0.281	−0.260	0.021	49.730	40.780	37.030	0.425
*Cyprinodon rubrofluviatilis*	NC009125	16501	831	0.260	0.294	0.171	0.274	−0.264	−0.026	54.160	41.090	45.940	0.471
*Fundulus olivaceus*	NC011380	16509	853	0.279	0.261	0.161	0.299	−0.236	−0.033	49.240	40.410	36.640	0.421

A, C, G, T, GCskew and ATskew refer to whole mitogenome content analysis. GC1, GC2, GC3 and Coding GC refer to the analysis of the mitochondrial coding-genes located in the H strand. See Additional file 4 for discriminated base composition values at first, second and third codon position of these 12 coding-genes located in the H strand

^a^ *K. marmoratus* CR1

^b^Annual species

Table 4 shows GenBank accession numbers, total length, CR length and the compositional properties of mitogenomes from *A. charrua*, *N. furzeri*, *K. marmoratus*, *A. panchax*, *C. rubrofluviatilis* and *F. olivaceus*. Regarding the *A. charrua* mitogenome G+C content is 37 %, while both GCskew (GCskew: (G-C)/(G+C)) and ATskew (ATskew: (A-T)/(A+T)) are negative, pointing to strand asymmetry in the nucleotide composition (excess of cytosine and thymine in the H strand, see below). It is worth noting that annual species present the lowest G+C content and GCskew, in spite of *K. marmoratus*, a sister taxon of *A. charrua*. In the case of ATskew, values are positive and negative among considered species, having *A. charrua* the highest asymmetry (−0.102). In fact, this relatively strong negative value is highly unusual in Actinopterygii and even Chordata [53]. Given the differences in length of the annotated control regions (Table 4) and the inherent difficulty to sequence this region, we hypothesized that their putative incompleteness in some of the species analyzed could have biased the above results. For this reason, we repeated the compositional analyses taking into account the sequences of the 12 protein-coding genes located in the H strand. The G+C content is again lower in both annual killifish, being the difference (as expected) larger in the first and third codon positions (Table 4 and Additional file 5). In order to determine if the lower G+C content in annual killifish is an ancestral or recently derivative character, we performed a sequence reconstruction of ancestral nodes with a joint-likelihood method in the codon-state space [26, 27]. Estimation of G+C content at the internal nodes of our reference topology (Additional file 1) confirms that the lower G+C content is a recently and independently derivative character state in both annual lineages (Additional file 6).

**Fig. 4:**
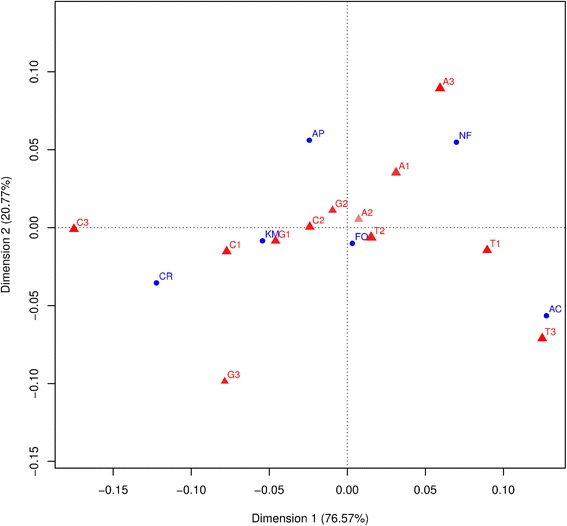
Correspondence analysis of nucleotide composition of all protein coding genes located in the H strand of all Cyprinodontiformes analyzed. Blue dots represent the species: AC (*A. charrua*), KM (*K. marmoratus*), NF (*N. furzeri*), AP (*A. panchax*), CR (*C. rubrofluviatilis*) and FO (*F. olivaceus*). Red triangles indicate the nucleotides at 1st, 2nd and 3rd codon position

Correspondence analyses of nucleotide composition of the coding-genes of the H strand (Fig. 4) and on global codon usage (Additional file 7) segregates annual killifishes from the remaining Cyprinodontiformes species. Codons rich in A and T at third position show the highest weight in this pattern, reflected in the first axis. The second axis segregates both aplocheiloid non-rivulids fishes (*N. furzeri* and *A. panchax*) from the remaining cyprinodontiforms and separates C from G and A from T. While organisms position in the first axis correlates with mitogenome G+C content (Spearman rank correlations were: GC: Spearman’s ρ = −1.00, p-value <0.05; GC3: Spearman’s ρ = −0.99, *p*-value <0.05 and GCskew: Spearman’s ρ = 0.90, *p*-value <0.05), second axis correlates to ATskew (ATskew: Spearman’s ρ = 0.93, *p*-vale <0.05). Both analyses revealed that annual species appear associated with an increase in A or T content in 1st and 3rd positions. Moreover, the second axis separates *A. charrua* from *N. furzeri*, the first species associated with A1 and A3 while the second appear richer in T1 and T3. When studying per gene codon usage considering only *A. charrua*, *K. marmoratus* and *N. furzeri*, there is a clear difference in per species codon usage, being both annual taxa more similar, in spite of the closer phylogenetic relationship between *A. charrua* and *K. marmoratus* (Additional file 8).

Furthermore, comparison of base usage among organisms shows an important difference of C over G at the three codon positions (Table 4 and Additional file 5), a fairly common characteristic [54]. However, only *A. charrua* displayed a net difference of T over A at 1st and 3rd positions. This feature could be explained by the fast growth and high metabolic rates present in annual fishes [10, 55]. These two processes involve multiple rounds of organelle replication per cell division with a concomitant increase of replication errors and of the concentrations of ROS in the mitochondria environment [56–59]. Reactive oxygen species promote GC to AT mutations through the deamination of cytosine and the oxidative conversion of guanine to 8-oxo-guanine. Hydrolytic deamination of cytosine or adenine and oxidation of guanine are among the described causes of mutational damage [60]. Given these sources of mutations and the pattern observed in *A. charrua*, an increase in the rate of hydrolytic deamination of cytosine in the H strand could be the main mechanism associated to the increase in thymine. In turn, the asymmetric pattern in base composition could be explained by the long-standing strand-displacement model of mtDNA replication: in this asymmetric process a portion of the H strand would remain a period of time in single strand state, which could lead to different mutational bias in H and L strands [61]. While this model of mtDNA replication has been challenged in recent years (reviewed in [62]), asymmetric trends in base composition have been confirmed [54].

Other various hypotheses have tried to explain the high adenine and thymine (AT) content of almost all mitochondrial DNAs [63]. One is that natural selection contributed to the high AT content of mitochondrial genomes, where selection for translational efficiency and accuracy shaped the nucleotide composition of codons in organelle genes, in some cases enriching the thymine content of synonymous sites [64]. Others postulated that AT richness is an adaptation for metabolic efficiency, due the increased energetic costs of producing C vs. T and G vs. A and the varying abundance of A/T vs. G/C nucleotides during organelle DNA synthesis [59, 65, 66]. As expected, codon usage pattern also translates into differences in amino acid usage (Additional file 9). While relative differences are small, there is a higher usage of phenylalanine in both rivulids, while both annual taxa share an increment of tyrosine, lysine and methionine usage.

### Selective constraints in coding regions

Variations in the subunits of the oxidative phosphorylation pathway have been linked to different life-history traits and environmental adaptations [67–70]. In a landscape of widespread strong purifying selection due to functional constraints, signals of positive selection have been reported for individual codon sites (or amino acids) of taxa known to have high energetic demanding lifestyles such as flying in bats [71] and unusual oxygen availability such as high altitude Caprini and subterranean octodontoid rodents [72, 73], among other conditions [70, 74, 75].

Given the annual life-style of *A. charrua* and *N. furzeri* we hypothesized that, in comparison to other cyprinodontiforms, they have been under a different selective regime and this could have left molecular signatures at the mitochondrial genome level. In fact, as explained above, both taxa present an independent decrease in their mitogenome G+C content that could be due to their fast growth and high metabolic rates. For many years codon models have been used to estimate the ratio of non-synonymous to synonymous substitution rate (dN/dS, often denoted as ω) and positive selection can be inferred whenever the estimated ratio dN/dS significantly exceeds one [76, 77]. The early codon models have been extended to permit the intensity of selection to vary among sites within a gene, among branches within a tree or both (branch-site models), greatly improving the power to detect positive selection. More recently a mixed effects model of evolution (MEME), a highly sensitive branch-site random effects phylogenetic method, was presented [29]. As it is capable of detecting episodic adaptation, as would be the case of the two annual lineages under study, we used MEME to search for evidence of positive directional selection in our mitochondrial codon sequences dataset.

As expected for their vital role in the oxidative phosphorylation, all 13 protein-coding genes are under strong purifying selection, with dN/dS values below 0.3 (Additional file 10). The ATPase6, ATPase8 and ND6 genes showed the highest dN/dS values, while components of the cytochrome oxidase complex (COI, COII and COIII) showed the lowest rates. These results are in perfect agreement with a previous comparative analysis in metazoans [53]. The analysis conducted with MEME [29] inferred four sites where some branches experienced a significant greater non-synonymous than synonymous substitution rate. These sites are: codon 121 of ATPase6; codon 47 of ATPase8; codon 369 of Cytb and codon 220 of ND2 (positions follow *A. charrua* annotation; Table 5). Molecular footprints of positive selection have been previously associated to mitochondrial ND2 in at least one work [70] and signatures of adaptation have been repeatedly detected in Cytb, ATPase6 and ATPase8 [70, 71, 73, 74, 78, 79]. Noteworthy, in three of these sites the episodic directional selection would have happened in the ancestral branch leading to rivulids (ATPase6, ATPase8 and ND2), while in the Cytb site it would have occurred in the tree branch leading to *N. furzeri* and *A. panchax* (Table 5). We did not find any significant evidence of sites showing episodic selection at both annual lineages. However, as MEME authors denote [29], it is difficult to accurately identify individual positively selected branches at an individual site, thus conclusions about specific lineages under selection in particular sites must be taken with caution.

**Table 5:** Codon sites and branches under episodic and pervasive selection inferred by mixed effects model of evolution (MEME [29])

Gene	Site	α	β-	β+	Pr[β=β+]	*p*-value	Putative branch under episodic selection	BEF	Syn subs	Non-syn subs
ATPase6	121	0.00	0.00	23.64	0.23	0.02	Branch leading to Rivulidae	704	0.25	2.75
ATPase8	47	0.05	0.05	96.16	0.12	0.05	Branch leading to Rivulidae	190	1.00	1.00
ND2	220	0.11	0.11	1.25	0.43	0.05	Branch leading to Rivulidae	31	0.00	2.00
Cytb	369	0.01	0.01	9.27	0.15	0.03	Branch leading to *N. furzeri* and *A. panchax*	75	1.00	2.00

*BEF* Bayes empirical factor, *Syn subs and non-syn subs* number of synonymous and non-synonymous substitutions inferred for the codon site, respectively

As explained, one cannot simultaneously infer the site and the branch subject to diversifying selection [29], the BS-REL model [30] was used in a complementary approach to analyze individual protein-coding genes, as well the concatenated “supergene”. No significant results were obtained for individual genes, but *A. charrua* and *N. furzeri* showed a small proportion of COI sites with ω values above 5 (*p*-values <0.1, data not shown). About the concatenated data set, the tree branch leading to the clade composed by *N. furzeri* and *A. panchax* showed evidence of strong directional selection (ω >>5; corrected *p*-value <<0.01) at about 1 % of the sites (out of a total of 3787 codon sites analyzed; Additional file 11). The branch of *N. furzeri* showed about 5.5 % of the sites under strong selection (ω >>5) but in this case is not statistically significant at the chosen 0.05 level (corrected p-value <0.1).

In summary, we were unable to identify elevated rates of adaptive evolution in annual species as expected by their different life traits and lower G+C mitochondrial genome content. For the analyses, we worked with only six orthologs sequences in each case, and it is known that such small alignments use to have low power for phylogenetic methods of adaptive sequence evolution [27]. However, it appears that at least *N. furzeri* could exhibit molecular signatures of adaptive evolution in case of a proper taxon sampling scenario (i.e. a broaden taxa sampling within Rivulidae, Nothobranchiidae and Aplocheilidae).

### *Austrolebias charrua* coding genes as phylogenetic molecular markers

Mitochondrial genes are some of the most popular and widely-utilized genetic markers in phylogenetic and phylogeographic studies [80]. As all genes reside in the same non-recombinant DNA molecule, their linked nature allows the assumption that they carry the same phylogenetic signal and share it with the entire mitogenome. However, as previously described, we found evidence of positive selection in the ATPase6, ATPase8, ND2 and Cytb genes and it is know that base composition changes at a different rate between lineages and genes affect adversely the efficiency of phylogenetic inference methods [54]. In this context, we assessed the performance of single mitochondrial protein-coding genes and a concatenated “supergene” at inferring evolutionary relationships throughout Cyprinodontiformes. This allowed us: i) to identify whether each protein-coding gene possesses the same phylogenetic signal and thus it is interchangeable; and ii) to distinguish how individual genes perform compared to the concatenated “supergene”. A phylogenetic inference was conducted for each gene and “supergene” in a maximum likelihood framework and each resulting gene tree visually compared to the reference species tree (Additional file 1). We did not try any mitochondrial specific DNA substitution models (e.g. GTR3 in [54]), thus we did not assessed putative inconsistencies due to base composition features.

**Fig. 5:**
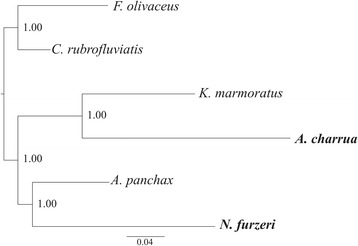
Maximum likelihood analysis of a concatenated alignment of all 13 protein-coding genes “supergene” without the 3rd codon position. Branch support was conducted with a Bayesian-like transformation of approximate likelihood ratio test. The nucleotide substitution model was TVM+G determined according to the best suited sequence substitution model selected by ModelGenerator v0.85 [35], following Bayesian information criterion

Regarding whole coding sequence alignments, the inferred relative positioning of the lineages was in agreement with the reference species tree only for Cytb, ND1 and ND6 gene trees, while the concatenated “supergene” did not recover the reference species topology (Additional file 12). This is concordant with previous analyses using Cytb gene to resolve molecular phylogenetic relationships among species of the genus *Austrolebias* [6–8]. Moreover, most phylogeographic studies in different species groups within *Austrolebias* were based on this gene [81–83]. When fast evolving third codon positions were removed from alignments, the “supergene” tree recovers our reference species phylogeny (Fig. 5) as well as the Cytb, ND4L and ND6 genes (Additional file 13). Therefore, third codon positions seem to be saturated in the aforementioned coding regions at intergeneric Cyprinodontiformes comparisons.

While our taxonomic sampling is reduced and far from being an exhaustive and proper sample of cyprinodontiforms lineages, evidence points out differences in the phylogenetic informativeness level among the mitochondrial protein-coding genes. Even the concatenated gene set did not appear to perform consistently either. Considering cost-effectiveness trade-offs, Cytb appears as a proper molecular marker to be chosen when studying phylogenetic relationships, at inter- and intraspecific levels in Cyprinodontiformes, as was frequently used by Murphy and Collier [84, 85] and García et al. [6, 7] as well as in other highly related Rivulidae genera [86].

## Conclusions

The mitogenome of *A. charrua* obtained in present work, represents the first one among Neotropical annual killifishes. It contains a gene arrangement and composition similar to most vertebrate mitogenomes. Among cyprinodontiforms, the mtDNA of *A. charrua* codes for the shortest tRNA-Cys without the D-loop arm, exhibits the lower G+C content, and the highest strand asymmetry. Our analyses showed that annual killifishes present an independent decrease in their mitogenome G+C content pointing to a change in the regimen of evolutionary forces that interact with the mitochondrial mutational bias.

## Additional files

Additional file 1:
**Phylogenetic relationships among some cyprinodontiforms families of fishes according to [** 23 **].** (PDF 122 kb) Additional file 2;
**Selected nucleotide substitution models after the third codon positions were removed from the codon alignments.** (PDF 7 kb) Additional file 3:
**Dotplot of AUS7_control region obtained with zpicture (** http://zpicture.dcode.org/ **).** Duplicated regions are of 180 bp (from 808 to 987) and 183 bp (from 970 to 1152) with a 91.67 % of identity. (PDF 59 kb) Additional file 4:
**Jalview visualization of a multiple sequence alignment of metazoan Cytb sequences [** 87 **].** *A. charrua* Cytb sequence was used as query for a blastp search of metazoan homolog sequences in the NCBI’s nr database. Downloaded amino acidic sequences were added to the cyprinodontiforms sequences used in this work and the whole dataset aligned with Muscle [24]. The alignment positions are coloured by percentage of identity and the column corresponding to *A. charrua* Cytb F 368 is highlighted with a red dash (column 375 in the present alignment). (PDF 1903 kb) Additional file 5:
**Base composition of mitochondrial coding-genes located in the H strand of all cyprinodontiforms compared in present work.** Values are discriminated according to first, second and third codon position. (PDF 16 kb) Additional file 6:
**Percentage of the G+C content considering the 12 protein-coding genes located in the H strand.** In green, values for the G+C content of the sequence reconstruction of ancestral nodes with a joint-likelihood method in the codon-state space. In red, G+C content of *A. charrua* and *N. furzeri* and in black, values of the remaining cyprinodontiforms mitogenomes analyzed. (PDF 472 kb) Additional file 7:
**Representation of the first two dimensions of the correspondence analysis (COA) performed on the global codon usage of the six species of cyprinodontiforms analyzed in this study.** Variables (codons) and cases (species) are plotted together. Blue dots represent the species: AC (*A. charrua*), KM (*K. marmoratus*), NF (*N. furzeri*), AP (*A. panchax*), CR (*C. rubrofluviatilis*) and FO (*F. olivaceus*). Red triangles correspond to codons. (PDF 668 kb) Additional file 8:
**Correspondence analysis of per gene codon usage considering only** ***A. charrua*** **(AC),** ***K. marmoratus*** **(KM) and** ***N. furzeri*** **(NF).** Blue dots correspond to per gene codon usage of *A. charrua*, red to *N. furzeri* and black to per gene codon usage of *K. marmoratus*. (PDF 545 kb) Additional file 9:
**Representation of the first two dimensions of the correspondence analysis (COA) performed on the amino acid frequencies of the six species of cyprinodontiforms analyzed.** Variables (amino acids) and cases (species) are plotted together. The amino acids are represented in the usual one-letter code (red triangles). Blue dots represent the species: AC (*A. charrua*), KM (*K. marmoratus*), NF (*N. furzeri*), AP (*A. panchax*), CR (*C. rubrofluviatilis*) and FO (*F. olivaceus*). (PDF 567 kb) Additional file 10:
**Non-synonymous to synonymous rate ratio of all mitochondrial protein-coding genes.** (PDF 410 kb) Additional file 11:
**BS-REL results for concatenated data set.** Node 1, 2 and 5 as in Additional file 1. (PDF 479 kb) Additional file 12:
**Maximum likelihood analysis of all protein-coding genes.** a) ATPase6; b) ATPase8; c) COI; d) COII; e) COIII; f) Cytb; g) ND1; h) ND2; i) ND3; j) ND4; k) ND4L; l) ND5; m) ND6 and n) concatenated “supergene”. (PDF 298 kb) Additional file 13:
**Maximum likelihood analysis of all protein-coding genes without the 3rd codon position.** a) ATPase6; b) ATPase8; c) COI; d) COII; e) COIII; f) Cytb; g) ND1; h) ND2; i) ND3; j) ND4; k) ND4L; l) ND5 and m) ND6. (PDF 619 kb)

## Abbreviations

AA: Amino acid
ATPase 6–8: ATP synthase subunits 6–8
AUS4, 5, 7: *Austrolebias charrua* individual 4, 5 and 7
COI-III: Cytochrome c oxidase subunits I-III
CR: Control region
Cytb: Cytochrome b
GTR+I+G: General time reversible model with invariable sites and gamma distribution
HKY+I+G: Hasegawa-Kishino-Yano model with invariable sites and gamma distribution
ML: Maximum likelihood
mtDNA: Mitochondrial DNA
ND1-6, 4L: NADH dehydrogenase subunits 1–6, 4L
PCR: Polymerase chain reaction
ROS: Reactive oxygen species
rRNA: Ribosomal RNA
tRNA: Transfer RNA
Ts: Transition
Tv: Transversion
