# Supplementary material for: First complete mitochondrial genome of the South American annual fish Austrolebias charrua (Cyprinodontiformes: Rivulidae): peculiar features among cyprinodontiforms mitogenomes
Source: BMC Genomics. 2015 Oct 28;16:879. doi: 10.1186/s12864-015-2090-3 (PMC4625726; doi:10.1186/s12864-015-2090-3)

Additional file 1: Phylogenetic relationships among some cyprinodontiforms families of fishes according to [23].

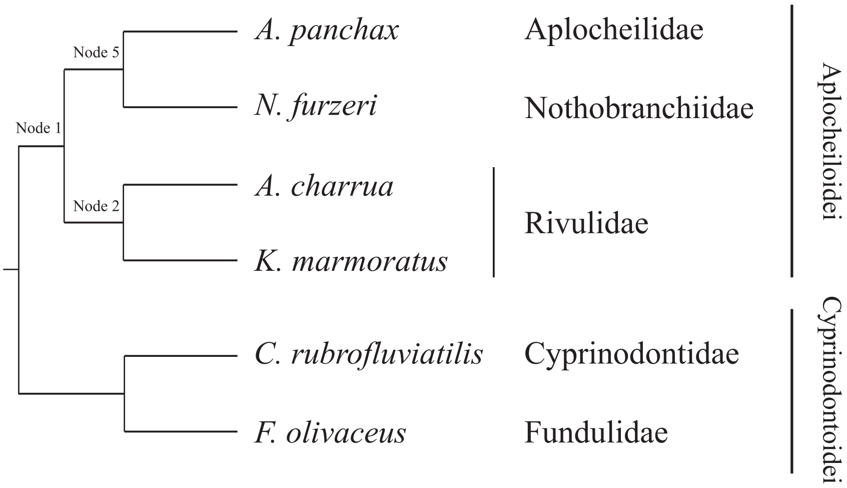

Supplement: Additional file 1: — Phylogenetic relationships among some cyprinodontiforms families of fishes according to [ 23 ]. (PDF 122 kb) [file 12864_2015_2090_MOESM1_ESM.pdf]
