# Supplementary material for: First complete mitochondrial genome of the South American annual fish Austrolebias charrua (Cyprinodontiformes: Rivulidae): peculiar features among cyprinodontiforms mitogenomes
Source: BMC Genomics. 2015 Oct 28;16:879. doi: 10.1186/s12864-015-2090-3 (PMC4625726; doi:10.1186/s12864-015-2090-3)
Supplement: Additional file 2; — Selected nucleotide substitution models after the third codon positions were removed from the codon alignments. (PDF 7 kb) [file 12864_2015_2090_MOESM2_ESM.pdf]

Additional file 2: Selected nucleotide substitution models after the third codon positions were removed from the codon alignments.

Gene	Nucleotide substitution model
ATPase6	HKY+G
ATPase8	HKY+I
Cytb	HKY+G
COI	TRN+I
COII	K80+G
COIII	HKY+I
ND1	HKY+G
ND2	TVM+G
ND3	HKY+G
ND4	TVM+G
ND4L	TrN+I
ND5	TVM+G
ND6	HKY+G
concatenated gene set	TVM+G
