# Supplementary material for: First complete mitochondrial genome of the South American annual fish Austrolebias charrua (Cyprinodontiformes: Rivulidae): peculiar features among cyprinodontiforms mitogenomes
Source: BMC Genomics. 2015 Oct 28;16:879. doi: 10.1186/s12864-015-2090-3 (PMC4625726; doi:10.1186/s12864-015-2090-3)

Additional file 3: Dotplot of AUS7_control region obtained with zpicture (http://zpicture.dcode.org/). Duplicated regions are of 180 bp (from 808 to 987) and 183 bp (from 970 to 1152) with a 91.67% of identity.

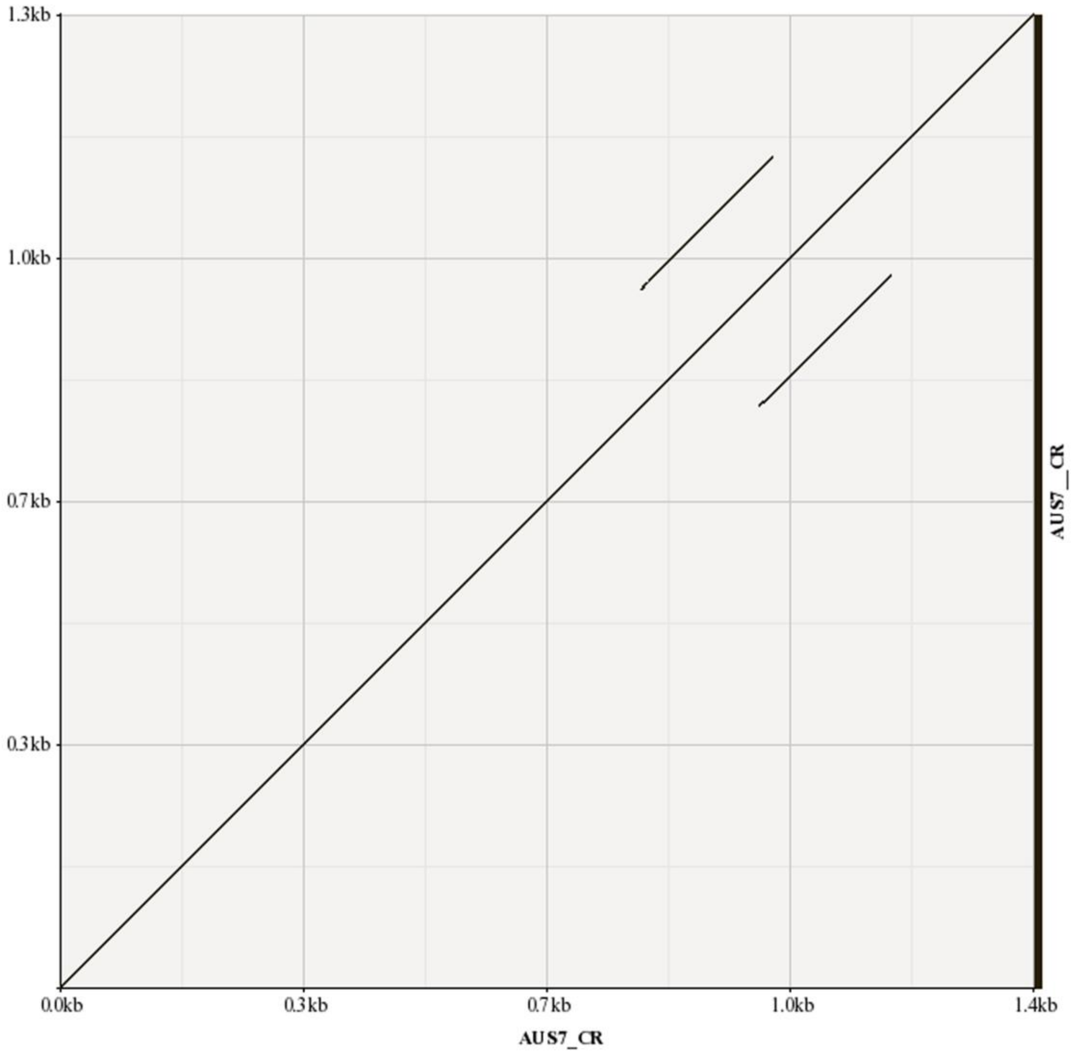

Supplement: Additional file 3: — Dotplot of AUS7_control region obtained with zpicture ( http://zpicture.dcode.org/ ). Duplicated regions are of 180 bp (from 808 to 987) and 183 bp (from 970 to 1152) with a 91.67 % of identity. (PDF 59 kb) [file 12864_2015_2090_MOESM3_ESM.pdf]
