# Supplementary material for: First complete mitochondrial genome of the South American annual fish Austrolebias charrua (Cyprinodontiformes: Rivulidae): peculiar features among cyprinodontiforms mitogenomes
Source: BMC Genomics. 2015 Oct 28;16:879. doi: 10.1186/s12864-015-2090-3 (PMC4625726; doi:10.1186/s12864-015-2090-3)

Additional file 4: Jalview visualization of a multiple sequence alignment of metazoan Cytb sequences [87]. *A. charrua* Cytb sequence was used as query for a blastp search of metazoan homolog sequences in the NCBI's nr database. Downloaded amino acidic sequences were added to the cyprinodontiforms sequences used in this work and the whole dataset aligned with Muscle [24]. The alignment positions are coloured by percentage of identity and the column corresponding to *A. charrua* Cytb F 368 is highlighted with a red dash (column 375 in the present alignment).

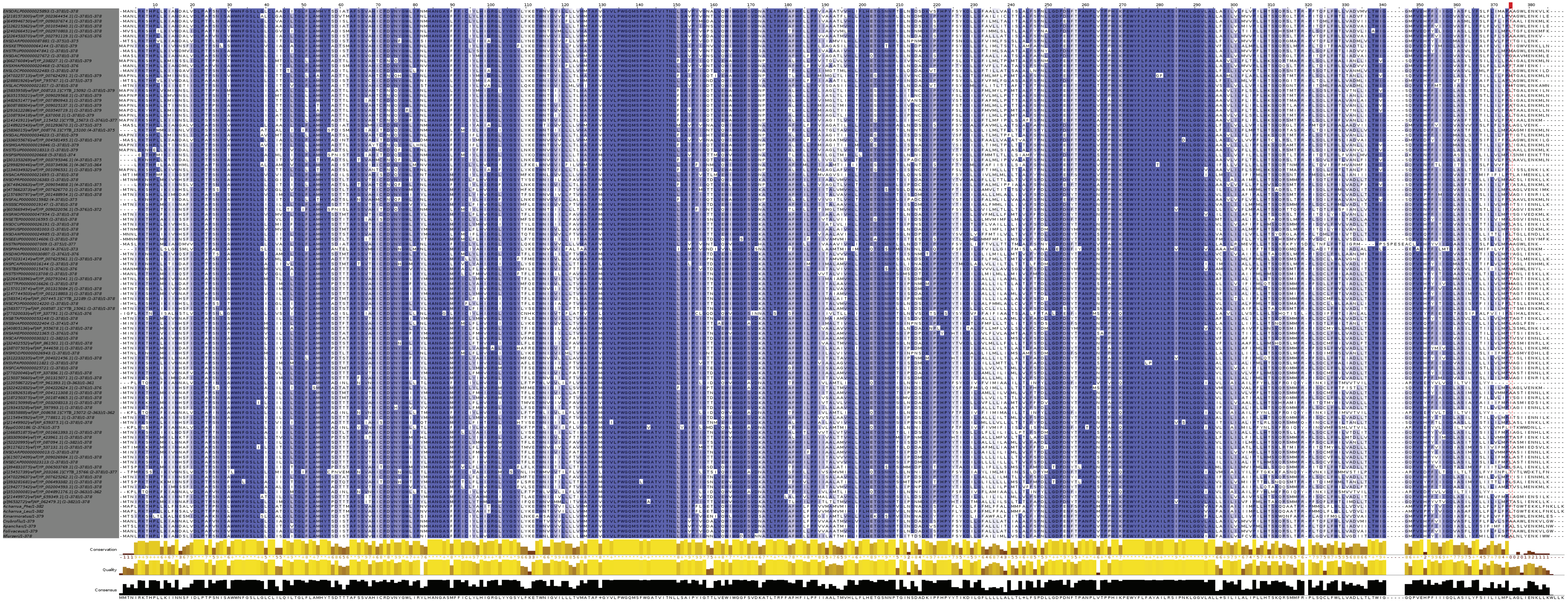

Supplement: Additional file 4: — Jalview visualization of a multiple sequence alignment of metazoan Cytb sequences [ 87 ]. A. charrua Cytb sequence was used as query for a blastp search of metazoan homolog sequences in the NCBI’s nr database. Downloaded amino acidic sequences were added to the cyprinodontiforms sequences used in this work and the whole dataset aligned with Muscle [24]. The alignment positions are coloured by percentage of identity and the column corresponding to A. charrua Cytb F 368 is highlighted with a red dash (column 375 in the present alignment). (PDF 1903 kb) [file 12864_2015_2090_MOESM4_ESM.pdf]
