# Supplementary material for: First complete mitochondrial genome of the South American annual fish Austrolebias charrua (Cyprinodontiformes: Rivulidae): peculiar features among cyprinodontiforms mitogenomes
Source: BMC Genomics. 2015 Oct 28;16:879. doi: 10.1186/s12864-015-2090-3 (PMC4625726; doi:10.1186/s12864-015-2090-3)
Supplement: Additional file 5: — Base composition of mitochondrial coding-genes located in the H strand of all cyprinodontiforms compared in present work. Values are discriminated according to first, second and third codon position. (PDF 16 kb) [file 12864_2015_2090_MOESM5_ESM.pdf]

Additional file 5: Base composition of mitochondrial coding-genes located in the H strand of all cyprinodontiforms compared in present work. Values are discriminated according to first, second and third codon position.

Species	A1	C1	G1	T1	A2	C2	G2	T2	A3	C3	G3	T3
Austrolebias charrua	0.267	0.231	0.219	0.283	0.186	0.262	0.125	0.428	0.320	0.213	0.075	0.393
Nothobranchius furzeri	0.295	0.228	0.220	0.257	0.191	0.265	0.129	0.415	0.382	0.246	0.066	0.305
Kryptolebias marmoratus	0.266	0.257	0.238	0.239	0.184	0.274	0.128	0.414	0.304	0.335	0.081	0.280
Aplocheilus panchax	0.270	0.261	0.236	0.233	0.184	0.276	0.132	0.409	0.362	0.308	0.062	0.267
Cyprinodon rubrofluviatilis	0.250	0.290	0.252	0.209	0.184	0.281	0.130	0.405	0.276	0.366	0.094	0.265
Fundulus olivaceus	0.258	0.251	0.242	0.249	0.185	0.276	0.128	0.411	0.323	0.291	0.075	0.310
