# Supplementary material for: First complete mitochondrial genome of the South American annual fish Austrolebias charrua (Cyprinodontiformes: Rivulidae): peculiar features among cyprinodontiforms mitogenomes
Source: BMC Genomics. 2015 Oct 28;16:879. doi: 10.1186/s12864-015-2090-3 (PMC4625726; doi:10.1186/s12864-015-2090-3)

Additional file 6: Percentage of the G+C content considering the 12 protein-coding genes located in the H strand. In green, values for the G+C content of the sequence reconstruction of ancestral nodes with a joint-likelihood method in the codon-state space. In red, G+C content of *A. charrua* and *N. furzeri* and in black, values of the remaining cyprinodontiforms mitogenomes analyzed.

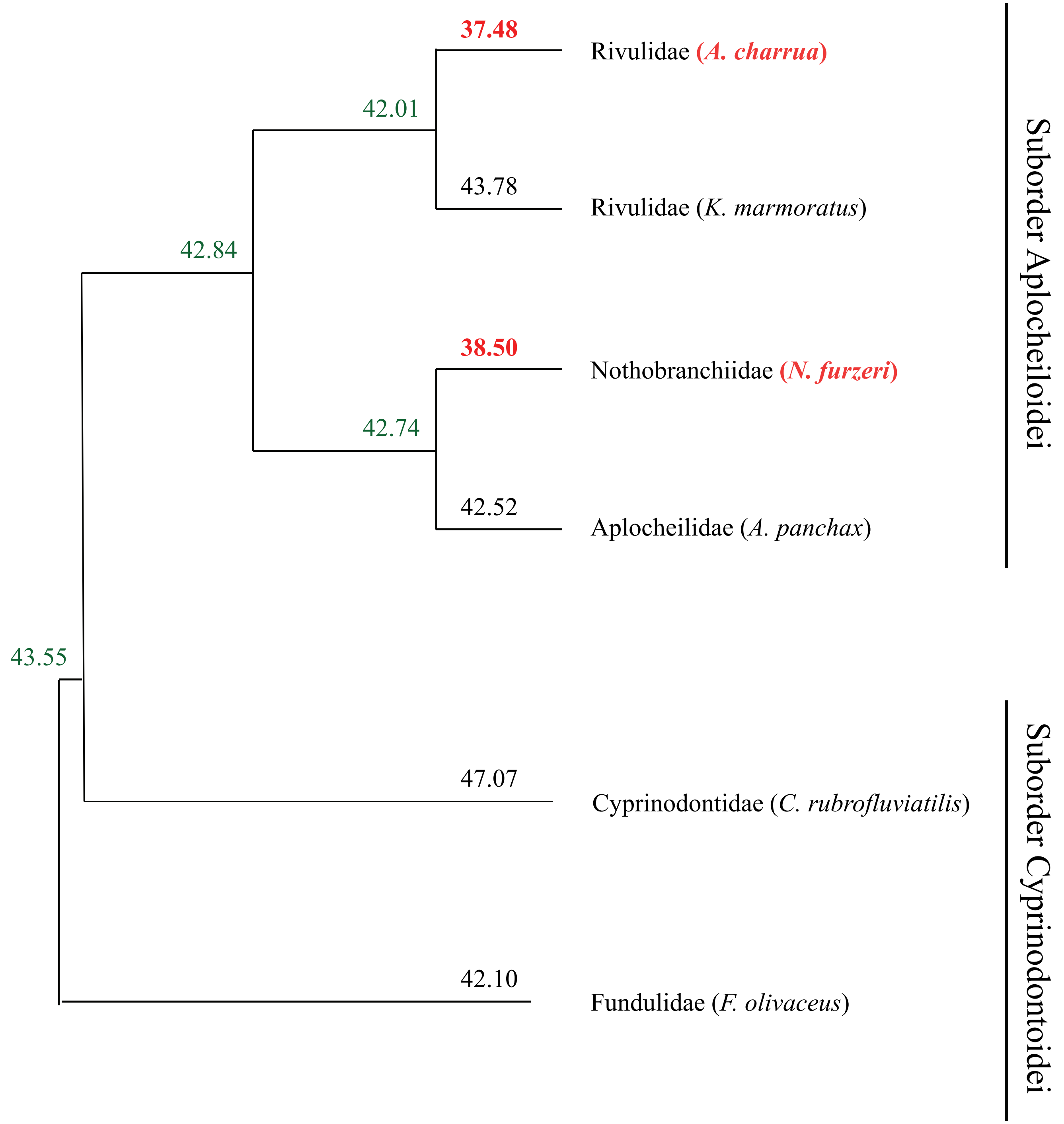

Supplement: Additional file 6: — Percentage of the G+C content considering the 12 protein-coding genes located in the H strand. In green, values for the G+C content of the sequence reconstruction of ancestral nodes with a joint-likelihood method in the codon-state space. In red, G+C content of A. charrua and N. furzeri and in black, values of the remaining cyprinodontiforms mitogenomes analyzed. (PDF 472 kb) [file 12864_2015_2090_MOESM6_ESM.pdf]
