# Supplementary material for: First complete mitochondrial genome of the South American annual fish Austrolebias charrua (Cyprinodontiformes: Rivulidae): peculiar features among cyprinodontiforms mitogenomes
Source: BMC Genomics. 2015 Oct 28;16:879. doi: 10.1186/s12864-015-2090-3 (PMC4625726; doi:10.1186/s12864-015-2090-3)

Additional file 7: Representation of the first two dimensions of the correspondence analysis (COA) performed on the global codon usage of the six species of cyprinodontiforms analyzed in this study. Variables (codons) and cases (species) are plotted together. Blue dots represent the species: AC (*A. charrua*), KM (*K. marmoratus*), NF (*N. furzeri*), AP (*A. panchax*), CR (*C. rubrofluviatilis*) and FO (*F. olivaceus*). Red triangles correspond to codons.

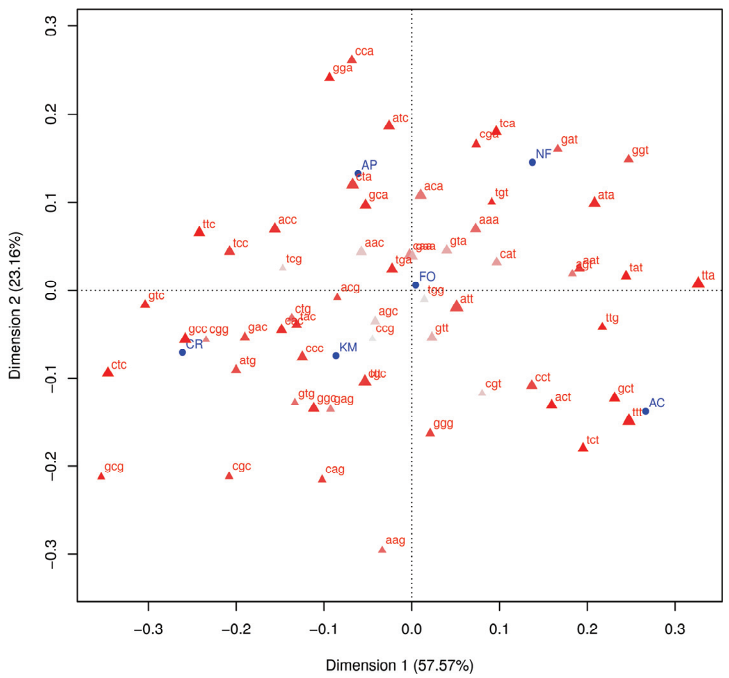

Supplement: Additional file 7: — Representation of the first two dimensions of the correspondence analysis (COA) performed on the global codon usage of the six species of cyprinodontiforms analyzed in this study. Variables (codons) and cases (species) are plotted together. Blue dots represent the species: AC (A. charrua), KM (K. marmoratus), NF (N. furzeri), AP (A. panchax), CR (C. rubrofluviatilis) and FO (F. olivaceus). Red triangles correspond to codons. (PDF 668 kb) [file 12864_2015_2090_MOESM7_ESM.pdf]
