# Supplementary material for: First complete mitochondrial genome of the South American annual fish Austrolebias charrua (Cyprinodontiformes: Rivulidae): peculiar features among cyprinodontiforms mitogenomes
Source: BMC Genomics. 2015 Oct 28;16:879. doi: 10.1186/s12864-015-2090-3 (PMC4625726; doi:10.1186/s12864-015-2090-3)

Additional file 8: Correspondence analysis of per gene codon usage considering only *A. charrua* (AC), *K. marmoratus* (KM) and *N. furzeri* (NF). Blue dots correspond to per gene codon usage of *A. charrua*, red to *N. furzeri* and black to per gene codon usage of *K. marmoratus*.

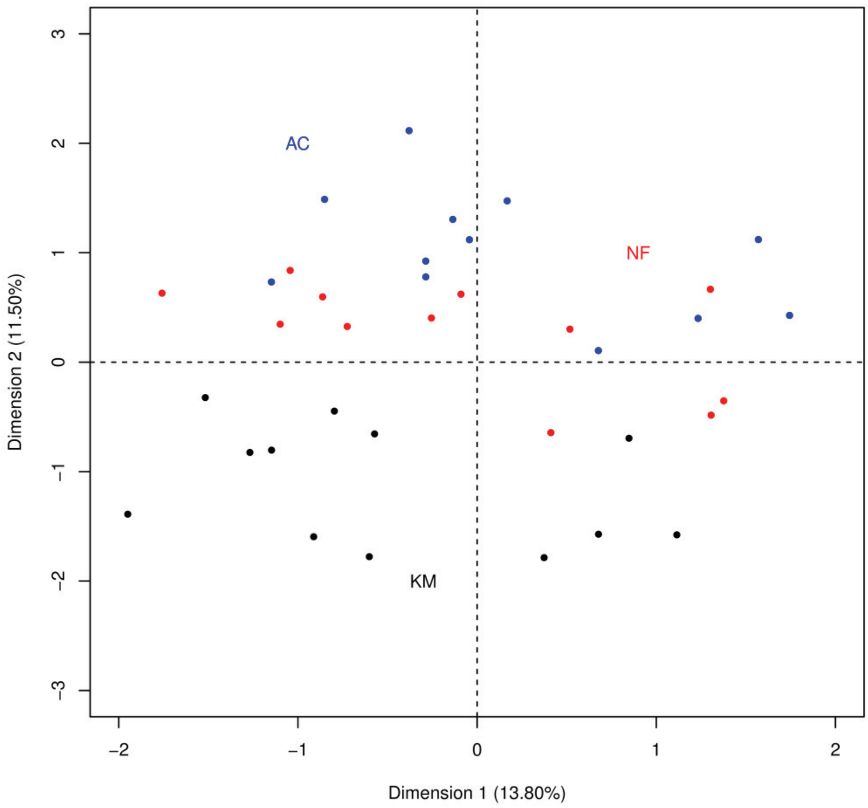

Supplement: Additional file 8: — Correspondence analysis of per gene codon usage considering only A. charrua (AC), K. marmoratus (KM) and N. furzeri (NF). Blue dots correspond to per gene codon usage of A. charrua, red to N. furzeri and black to per gene codon usage of K. marmoratus. (PDF 545 kb) [file 12864_2015_2090_MOESM8_ESM.pdf]
