# Supplementary material for: First complete mitochondrial genome of the South American annual fish Austrolebias charrua (Cyprinodontiformes: Rivulidae): peculiar features among cyprinodontiforms mitogenomes
Source: BMC Genomics. 2015 Oct 28;16:879. doi: 10.1186/s12864-015-2090-3 (PMC4625726; doi:10.1186/s12864-015-2090-3)

Additional file 9: Representation of the first two dimensions of the correspondence analysis (COA) performed on the amino acid frequencies of the six species of cyprinodontiforms analyzed. Variables (amino acids) and cases (species) are plotted together. The amino acids are represented in the usual one-letter code (red triangles). Blue dots represent the species: AC (*A. charrua*), KM (*K. marmoratus*), NF (*N. furzeri*), AP (*A. panchax*), CR (*C. rubrofluviatilis*) and FO (*F. olivaceus*).

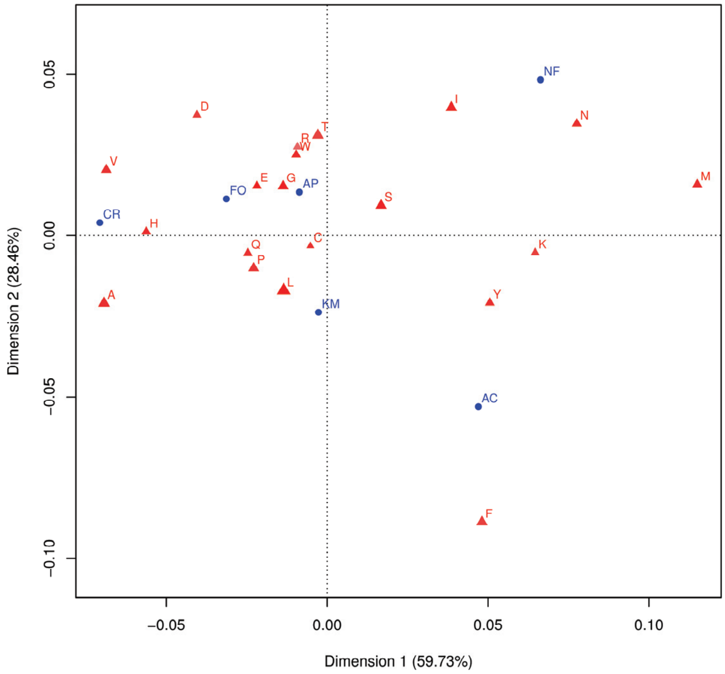

Supplement: Additional file 9: — Representation of the first two dimensions of the correspondence analysis (COA) performed on the amino acid frequencies of the six species of cyprinodontiforms analyzed. Variables (amino acids) and cases (species) are plotted together. The amino acids are represented in the usual one-letter code (red triangles). Blue dots represent the species: AC (A. charrua), KM (K. marmoratus), NF (N. furzeri), AP (A. panchax), CR (C. rubrofluviatilis) and FO (F. olivaceus). (PDF 567 kb) [file 12864_2015_2090_MOESM9_ESM.pdf]
