# Supplementary material for: First complete mitochondrial genome of the South American annual fish Austrolebias charrua (Cyprinodontiformes: Rivulidae): peculiar features among cyprinodontiforms mitogenomes
Source: BMC Genomics. 2015 Oct 28;16:879. doi: 10.1186/s12864-015-2090-3 (PMC4625726; doi:10.1186/s12864-015-2090-3)

Additional file 10: Non-synonymous to synonymous rate ratio of all mitochondrial protein-coding genes.

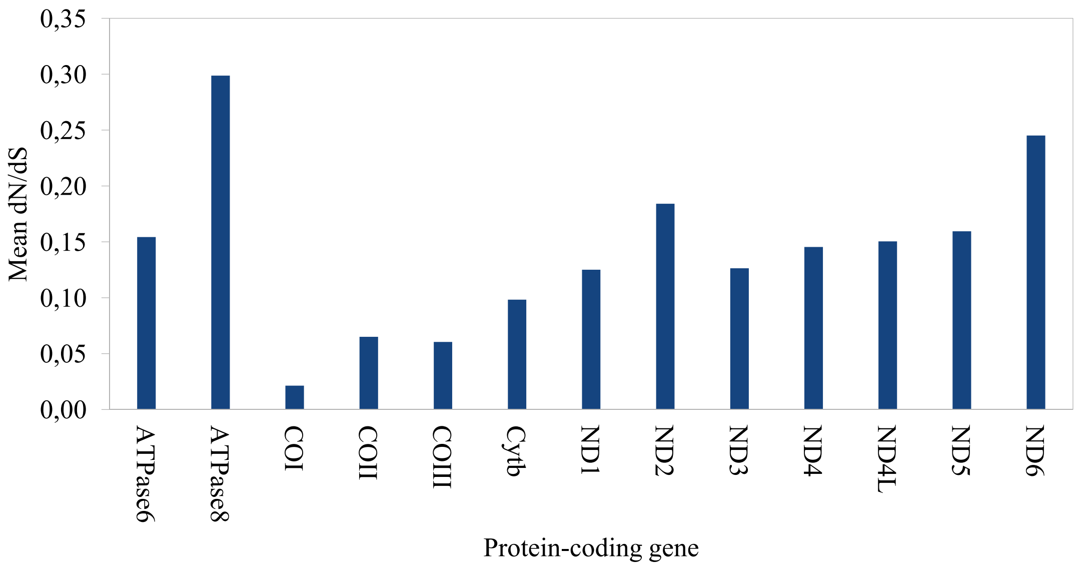

Supplement: Additional file 10: — Non-synonymous to synonymous rate ratio of all mitochondrial protein-coding genes. (PDF 410 kb) [file 12864_2015_2090_MOESM10_ESM.pdf]
