# Supplementary material for: First complete mitochondrial genome of the South American annual fish Austrolebias charrua (Cyprinodontiformes: Rivulidae): peculiar features among cyprinodontiforms mitogenomes
Source: BMC Genomics. 2015 Oct 28;16:879. doi: 10.1186/s12864-015-2090-3 (PMC4625726; doi:10.1186/s12864-015-2090-3)
Supplement: Additional file 11: — BS-REL results for concatenated data set. Node 1, 2 and 5 as in Additional file 1. (PDF 479 kb) [file 12864_2015_2090_MOESM11_ESM.pdf]

Additional file 11: BS-REL results for concatenated data set. Node 1, 2 and 5 as in Additional file 1.

Branch	Mean omega	omega1	p1	omega2	p2	omega3	p3	LRT	p-value	Corrected p-value
Node1	0.0593	0.0005	0.9401	0.9665	0	1.8262	0.0599	1.7664	0.0919	0.5515
Node2	0.2933	0.2879	0.9532	0	0.0193	3333.2900	0.0275	3.0750	0.0398	0.2783
Austrolebias charrua	0.0631	0.0033	0.7335	1	0.0803	0.2901	0.1861	0	1	1
Kryptolebias marmoratus	0.0719	0.0116	0.8287	0.0864	0.0258	0.7322	0.1455	0	1	1
Node5	10	1	0.9754	1	0.0139	2448.5400	0.0106	15.6262	0	0.0003
Nothobranchius furzeri	0.0622	0.0158	0.7549	0.2408	0.1882	444.0450	0.0570	6.0841	0.0068	0.0546
Aplocheilus panchax	0.0364	0.0162	0.9039	1	0.0553	0.0157	0.0408	0	1	1
Cyprinodon rubrofluviatilis	0.0314	0.0000	0.8957	0.3433	0.1000	1187.7800	0.0042	1.4557	0.1138	0.5690
Fundulus olivaceus	0.0411	0.0000	0.8472	0.3047	0.0274	0.4954	0.1255	0	1	1
