# Supplementary material for: First complete mitochondrial genome of the South American annual fish Austrolebias charrua (Cyprinodontiformes: Rivulidae): peculiar features among cyprinodontiforms mitogenomes
Source: BMC Genomics. 2015 Oct 28;16:879. doi: 10.1186/s12864-015-2090-3 (PMC4625726; doi:10.1186/s12864-015-2090-3)
Supplement: Additional file 12: — Maximum likelihood analysis of all protein-coding genes. a) ATPase6; b) ATPase8; c) COI; d) COII; e) COIII; f) Cytb; g) ND1; h) ND2; i) ND3; j) ND4; k) ND4L; l) ND5; m) ND6 and n) concatenated “supergene”. (PDF 298 kb) [file 12864_2015_2090_MOESM12_ESM.pdf]

Additional file 12: Maximum likelihood analysis of all protein-coding genes. a) ATPase6; b) ATPase8; c) COI; d) COII; e) COIII; f) Cytb; g) ND1; h) ND2; i) ND3; j) ND4; k) ND4L; l) ND5; m) ND6 and n) concatenated "supergene".

a)

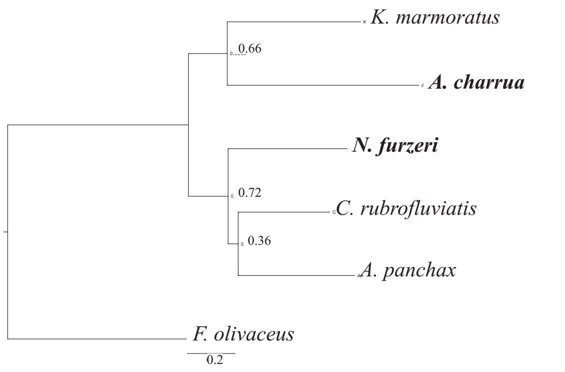

b)

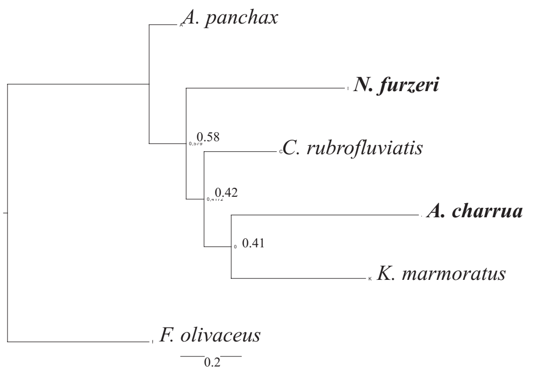

c)

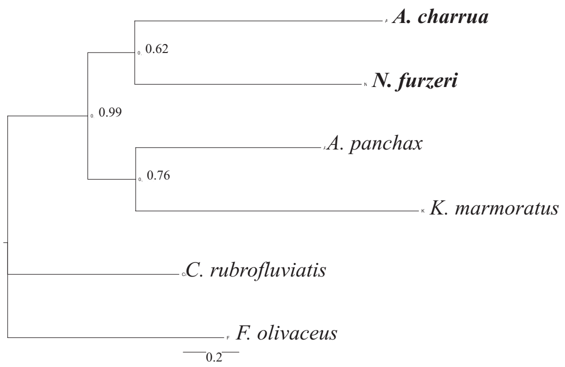

d)

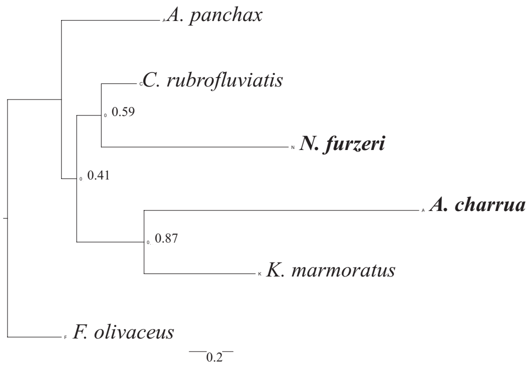

e)

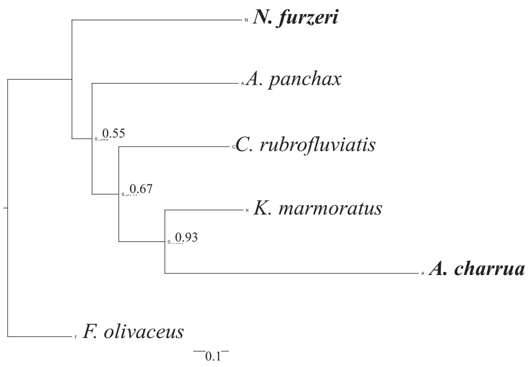

f)

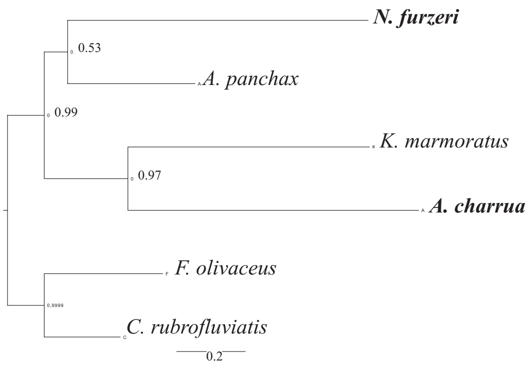

g)

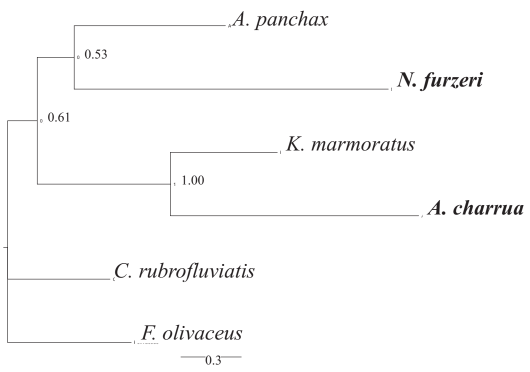

h)

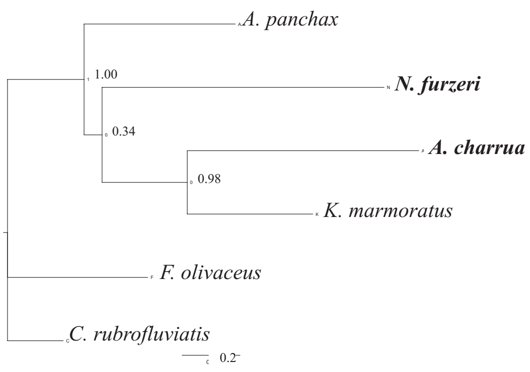

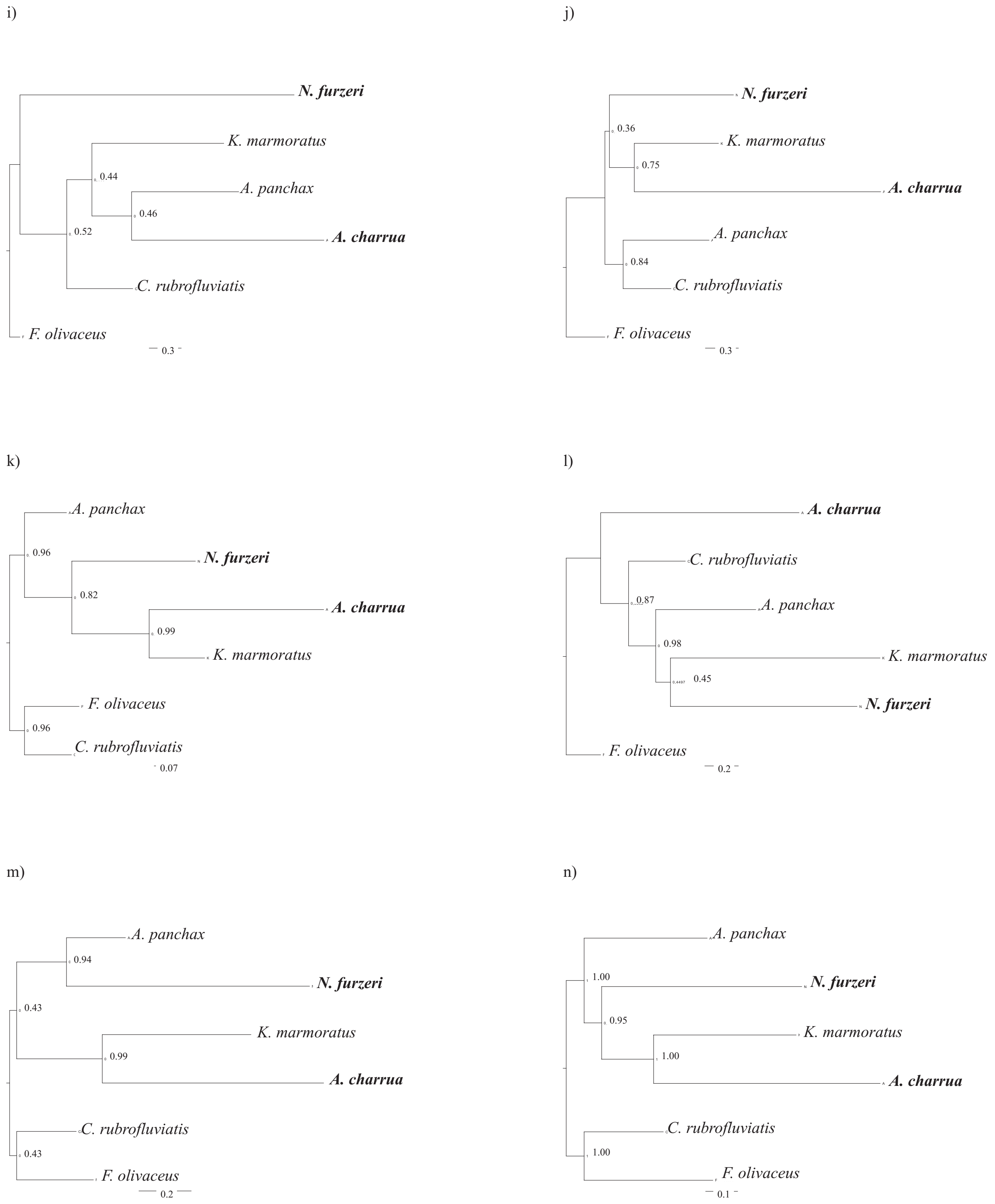

i)
N. furzeri
K. marmoratus
0.44
A. panchax
0.46
0.52
A. charrua
C. rubrofluviatis
F. olivaceus
0.3
j)
N. furzeri
0.36
K. marmoratus
0.75
A. charrua
A. panchax
0.84
C. rubrofluviatis
F. olivaceus
0.3
k)
A. panchax
0.96
N. furzeri
0.82
A. charrua
0.99
K. marmoratus
F. olivaceus
0.96
C. rubrofluviatis
0.07
l)
A. charrua
C. rubrofluviatis
0.87
A. panchax
0.98
K. marmoratus
0.45
N. furzeri
F. olivaceus
0.2
m)
A. panchax
0.94
N. furzeri
0.43
K. marmoratus
0.99
A. charrua
C. rubrofluviatis
0.43
F. olivaceus
0.2
n)
A. panchax
1.00
N. furzeri
0.95
K. marmoratus
1.00
A. charrua
C. rubrofluviatis
1.00
F. olivaceus
0.1
