# Supplementary material for: First complete mitochondrial genome of the South American annual fish Austrolebias charrua (Cyprinodontiformes: Rivulidae): peculiar features among cyprinodontiforms mitogenomes
Source: BMC Genomics. 2015 Oct 28;16:879. doi: 10.1186/s12864-015-2090-3 (PMC4625726; doi:10.1186/s12864-015-2090-3)
Supplement: Additional file 13: — Maximum likelihood analysis of all protein-coding genes without the 3rd codon position. a) ATPase6; b) ATPase8; c) COI; d) COII; e) COIII; f) Cytb; g) ND1; h) ND2; i) ND3; j) ND4; k) ND4L; l) ND5 and m) ND6. (PDF 619 kb) [file 12864_2015_2090_MOESM13_ESM.pdf]

Additional file 13: Maximum likelihood analysis of all protein-coding genes without the 3rd codon position. a) ATPase6; b) ATPase8; c) COI; d) COII; e) COIII; f) Cytb; g) ND1; h) ND2; i) ND3; j) ND4; k) ND4L; l) Nd5 and m) ND6.

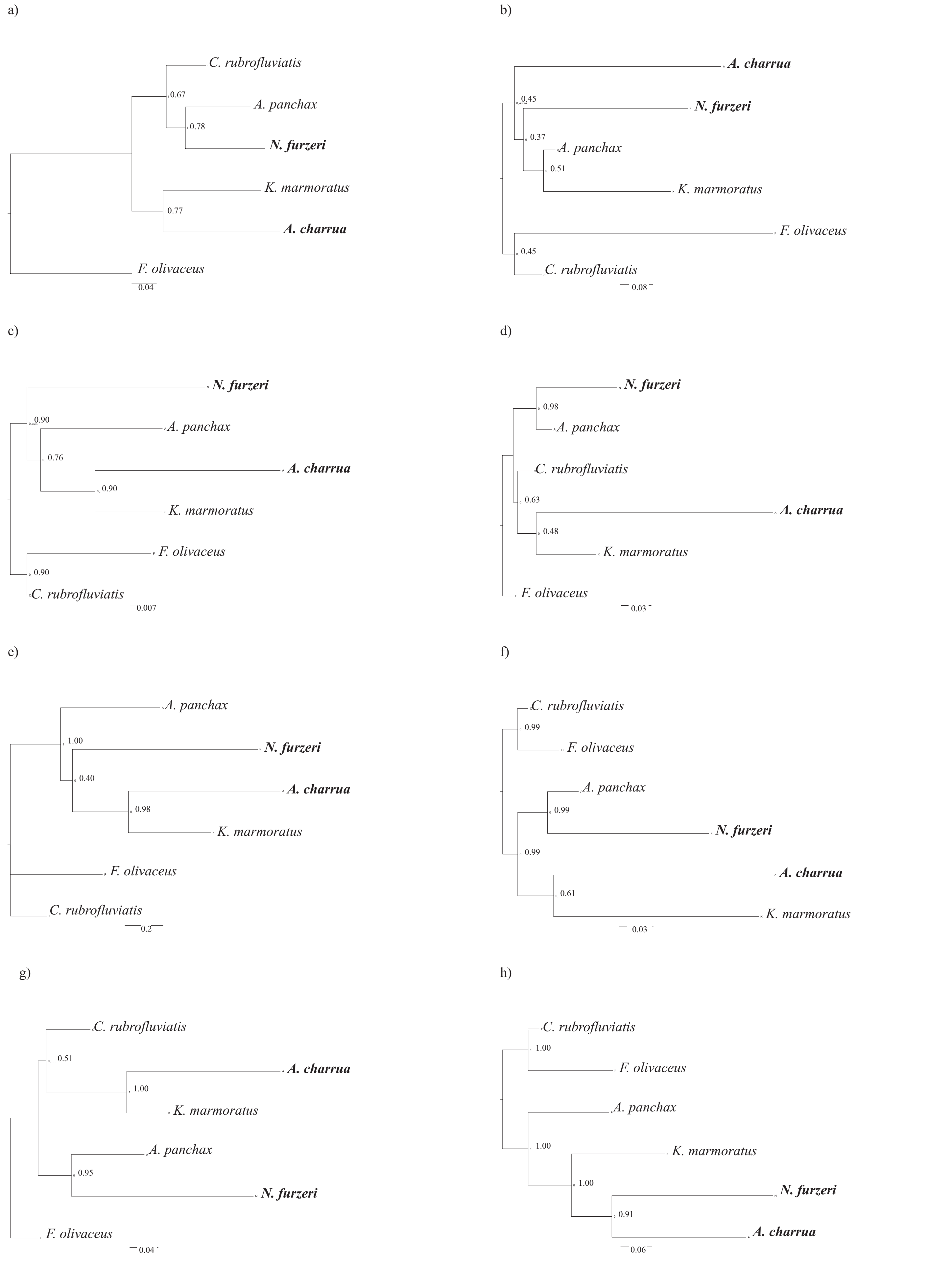

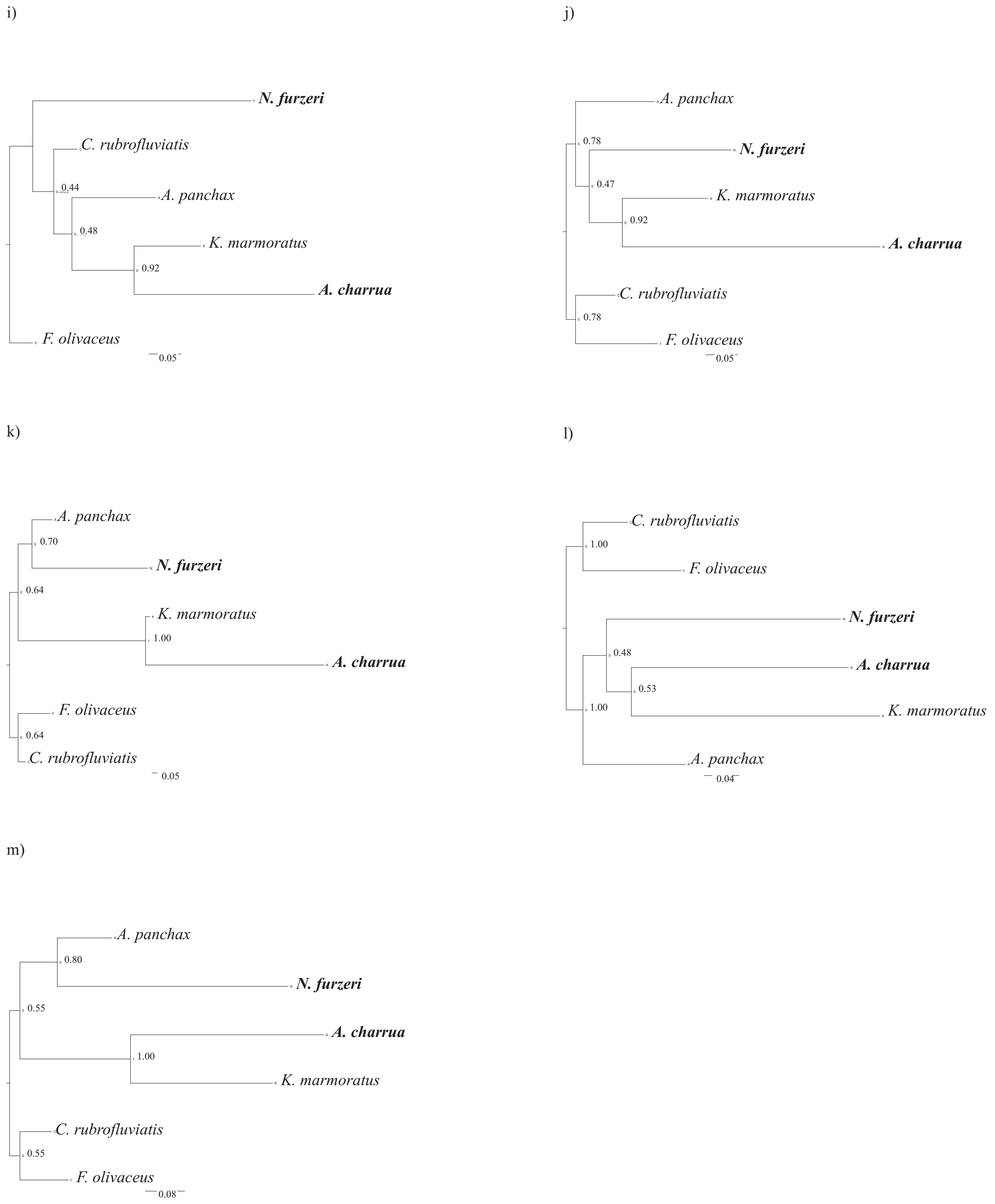

i)
N. furzeri
C. rubrofluviatis
0.44
A. panchax
0.48
K. marmoratus
0.92
A. charrua
F. olivaceus
0.05
j)
A. panchax
0.78
N. furzeri
0.47
K. marmoratus
0.92
A. charrua
C. rubrofluviatis
0.78
F. olivaceus
0.05
k)
A. panchax
0.70
N. furzeri
0.64
K. marmoratus
1.00
A. charrua
F. olivaceus
0.64
C. rubrofluviatis
0.05
l)
C. rubrofluviatis
1.00
F. olivaceus
N. furzeri
0.48
A. charrua
0.53
1.00
K. marmoratus
A. panchax
0.04
m)
A. panchax
0.80
N. furzeri
0.55
A. charrua
1.00
K. marmoratus
C. rubrofluviatis
0.55
F. olivaceus
0.08
